# Isothermal Strand Displacement Amplification for Chemical Sensing: From Molecular Design to Integrated and Intelligent Platforms

**DOI:** 10.3390/bios16100569

**Published:** 2026-10-10

**Authors:** Chia-Chun Lee, Wei-Ting Hsueh, Chin-Chung Tseng, Lung-Ming Fu

**Affiliations:** 1 Division of Nephrology, Department of Internal Medicine, National Cheng Kung University Hospital, College of Medicine, National Cheng Kung University, Tainan 704, Taiwan; n045740@mail.ncku.edu.tw (C.-C.L.); chinchun@mail.ncku.edu.tw (C.-C.T.); 2 Institute of Clinical Medicine, College of Medicine, National Cheng Kung University, Tainan 704, Taiwan; 3 Department of Oncology, National Cheng Kung University Hospital, College of Medicine, National Cheng Kung University, Tainan 704, Taiwan; seine@mail.ncku.edu.tw; 4 Department of Engineering Science, National Cheng Kung University, Tainan 701, Taiwan

**Keywords:** strand displacement amplification (SDA), isothermal amplification, biosensing, microfluidics, sample-to-answer analysis

## Abstract

Strand displacement amplification (SDA) provides a versatile isothermal framework for chemical and biosensing, coupling polymerase-driven strand displacement with programmable nucleic-acid signal generation. Its analytical scope now extends beyond direct nucleic-acid detection because aptamers, DNAzymes, antibodies, and responsive probes can convert diverse recognition events into amplifiable DNA triggers. This review develops a unified view of SDA from molecular design to practical implementation, with particular emphasis on programmable amplicon generation, recognition-to-DNA transduction, interfacial confinement, cascaded amplification, and CRISPR coupling. The review follows SDA across biomedical, food, and environmental applications, including RNA biomarkers, liquid biopsy, genetic and epigenetic targets, proteins, enzymes, small molecules, and viruses. It also considers how these assays are being transferred into microfluidic, paper-based, lateral-flow, self-powered, regenerative, and intelligent systems. The important distinction is that analytical sensitivity does not necessarily predict practical performance. Extremely low detection limits often depend on cascaded processing beyond SDA itself, whereas real samples expose weaknesses in matrix tolerance, background suppression, reagent stability, fluid control, assay duration, and manufacturing reproducibility. Comparison with commercially established isothermal methods further shows that wider adoption will depend on converting flexible molecular chemistry into robust, standardized, sample-to-answer systems.

## 1. Introduction

Rapid molecular analysis becomes most valuable when reliable measurements can be obtained close to the point of need rather than exclusively in centralized laboratories. Nucleic acids are particularly informative analytical targets because genomic DNA, messenger RNA, microRNA, long non-coding RNA, and pathogen-derived sequences carry highly specific molecular signatures associated with disease and infection [1,2,3,4]. The analytical reach can be extended to proteins, enzymes, extracellular vesicles, toxins, pesticides, and metal ions when aptamers, DNAzymes, antibodies, or responsive nucleic-acid probes translate target recognition into an amplifiable DNA output [2,5,6]. This capability is particularly important at trace concentrations, where target signals must be distinguished from the chemically complex backgrounds of blood, food, wastewater, and plant-derived samples [4]. Established analytical methods differ substantially in both capability and implementation. PCR and qPCR provide sensitive sequence-specific detection but depend on thermal cycling, whereas sequencing and chromatography generally require more specialized instrumentation and analytical infrastructure [7,8,9]. ELISA and lateral-flow assays illustrate how differently immunoassays can be deployed. The former supports quantitative laboratory analysis; the latter allows rapid testing with little instrumentation near the point of need. Which format is appropriate depends on the analyte, matrix, sensitivity, quantitative needs, and testing environment.

SDA can be traced back to 1992, when Walker and colleagues [10] demonstrated that DNA amplification could proceed continuously at 37 °C without repeated thermal cycling. Their design combined HincII-mediated nicking with exonuclease-deficient Klenow polymerase, whose extension activity displaced the downstream DNA strand and sustained the amplification process. The initial scheme relied on restriction cleavage and heat denaturation to generate amplification-ready targets; a subsequent formulation used strand-displacing polymerase activity to generate the required target ends without prior restriction digestion. Further work brought SDA into multiplex formats and subsequently adapted the reaction for thermophilic operation. Thermophilic SDA technology combines a thermostable restriction endonuclease with a strand-displacement polymerase at approximately 60 °C, significantly accelerating amplification while reducing nonspecific background. These early advances established a development trajectory that remains relevant today: simplifying target preparation, shortening reaction time, improving specificity, and making single-temperature amplification more robust.

Isothermal amplification encompasses several mechanistically distinct strategies, many of which reduce the thermal and instrumental demands associated with conventional PCR. These shared advantages alone do not explain the particular relevance of SDA to chemical sensing. What distinguishes SDA in this context is the close link between amplification and signal programming. Polymerase extension releases strands with defined sequences, while nicking, cleavage, or primer regeneration renews productive templates and keeps the amplification process running [4,11,12].

What matters analytically is not strand displacement alone, but what can be done with the released strands. Designed sequences may serve as primers, catalytic triggers, CRISPR activators, structural inputs, or reporter generators, creating a direct route from target recognition to molecular processing. This is especially useful when non-nucleic-acid targets, including proteins, enzymes, vesicles, and small molecules, are converted into DNA signals. What makes SDA relevant here is not simply its isothermal chemistry, but the way strand generation can be linked directly with amplification and downstream signal routing. The benefit is conditional, however: enzyme dependence, sequence constraints, background amplification, and leakage can all limit assay performance.

In SDA, DNA synthesis proceeds together with strand displacement, as the polymerase extends a primer and pushes the downstream strand away from the template. In the classical scheme, inner primers introduce the sequence required for nicking or restriction enzyme recognition, whereas outer primers promote formation of displaced strands that re-enter the amplification cycle. Nicking within the recognition sequence generates a free 3′ terminus for direct polymerase extension. As the new strand grows, the DNA lying downstream is peeled away and the recognition sequence is rebuilt, making the template available for another nicking event. The displaced DNA is not merely a reaction product; it becomes additional amplification material, so the process quickly multiplies the amount of target-derived DNA [12].

The comparison with enzyme-free strand-displacement circuits is intended to mark a mechanistic boundary, not a performance hierarchy. Catalytic hairpin assembly (CHA), hybridization chain reaction (HCR), toehold-mediated exchange, and related hybridization networks amplify signals through strand interactions without requiring polymerase-mediated strand synthesis. In SDA, by contrast, strand displacement is coupled directly to enzymatic extension and strand regeneration, producing new DNA strands that can re-enter the amplification cycle or serve as programmable inputs to downstream reactions [13,14]. This boundary also defines the review scope: SDA must provide the amplification core, while downstream CHA, HCR, CRISPR cleavage, or DNA-nanostructure assembly serves as signal processing.

This mechanistic distinction is also important when comparing SDA with PCR. PCR achieves highly established sequence amplification through repeated thermal denaturation, annealing, and extension, whereas SDA replaces repetitive thermal strand separation during amplification with enzymatic nicking and polymerase-driven displacement. The resulting lower thermal burden is advantageous for compact analytical hardware, although primer architecture, enzyme compatibility, background amplification, and reaction leakage remain important design constraints [15]. A side-by-side view of SDA and PCR is given in Table 1, covering how amplification proceeds, the temperatures and instruments required, typical assay times and applications, practical constraints, and their use in integrated sensing platforms [4,7,8,9,10,11,12,15].

**Figure 1:**
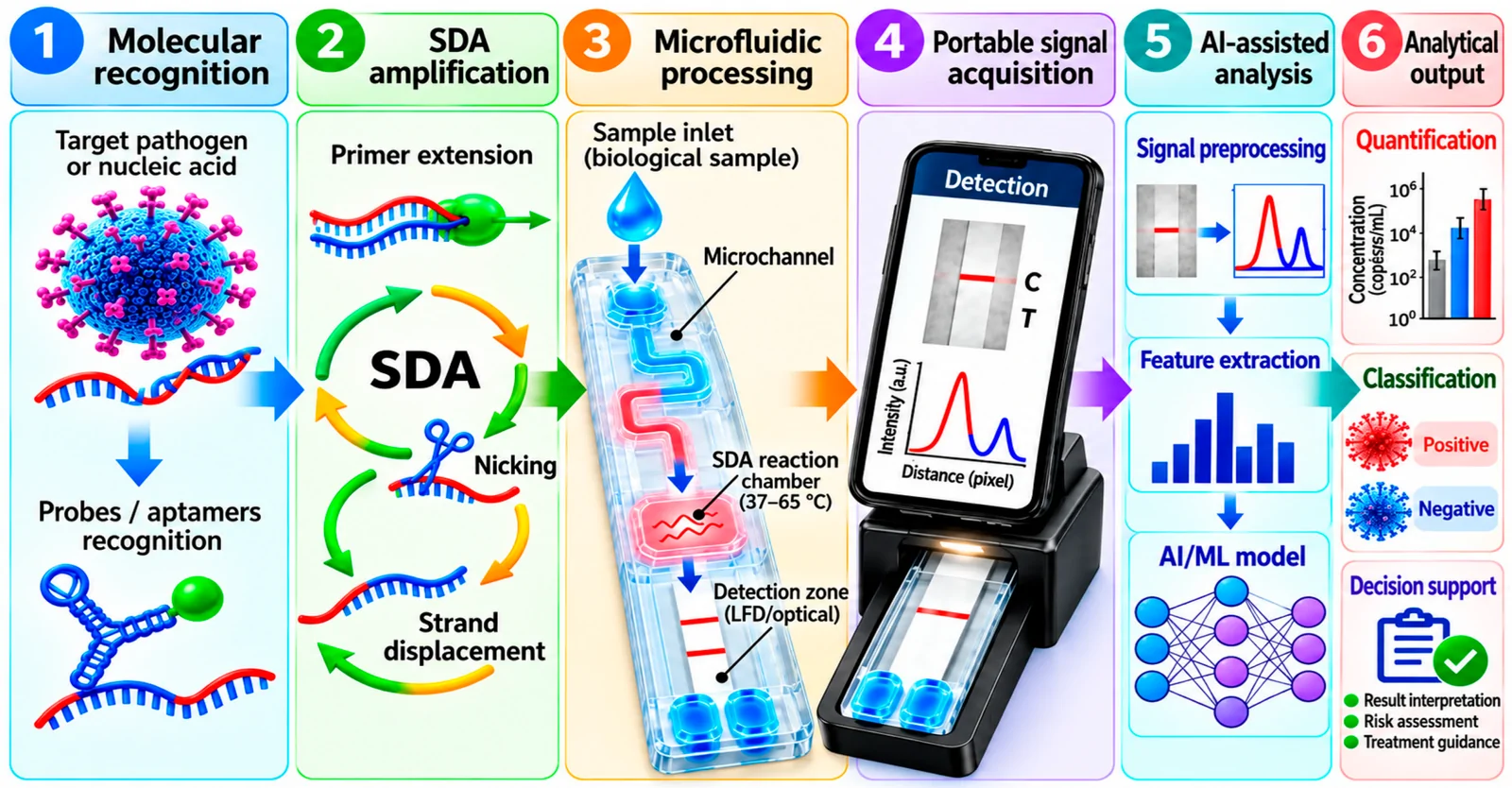
Overview of the review framework, linking SDA molecular design with biomedical, food/environmental applications, integrated sensing, AI-assisted interpretation, and translational considerations.

Microfluidics provides a natural environment for exploiting these characteristics. Small reaction chambers reduce reagent consumption and transport distances while allowing sample metering, target capture, amplification, washing, separation, and detection to be arranged within a controlled analytical sequence [16]. SDA products can subsequently be coupled to electrochemical, fluorescence, electrochemiluminescent (ECL), surface-enhanced Raman scattering (SERS), colorimetric, electrophoretic, or CRISPR-assisted readouts, enabling amplification chemistry to be separated conceptually from signal transduction [17,18]. Recent microchip electrophoresis and microfluidic SDA systems further illustrate how amplification and readout can be spatially coordinated without introducing thermal cycling as the dominant hardware requirement [19,20]. Such integration is more relevant to practical chemical sensing than sensitivity alone because it determines reagent handling, contamination control, operator intervention, and ultimately the feasibility of a sample-to-answer operation. Digital interpretation extends this integration beyond signal acquisition by converting amplified optical or electrochemical responses into quantitative or categorical analytical outputs. Figure 1 provides a conceptual roadmap of the review, linking molecular recognition and SDA with sample processing, portable signal acquisition, AI-assisted interpretation, and analytical output across biomedical, food/environmental, integrated-platform, and translational perspectives. The acquired optical or electrochemical signals can then enter an AI/ML layer for feature extraction, quantitative analysis, classification, and decision support.

Analytical sensitivity, however, should not be treated as a sufficient measure of translational performance. Many nucleic-acid biosensors report remarkably low detection limits using synthetic targets or carefully controlled matrices, conditions that do not necessarily predict behavior in clinical, food, or environmental samples [20,21,22]. Once SDA is taken beyond controlled laboratory settings, practical performance is shaped less by the headline detection limit than by recovery efficiency, matrix effects, surface fouling, enzyme robustness, background amplification, manufacturing consistency, and the time required to complete the assay [20,21,22]. Accordingly, the central question is not simply how far SDA can lower a detection threshold, but whether recognition, amplification, fluid handling, transduction, and data interpretation can operate reproducibly within the same analytical workflow.

SDA is considered here as a chemical-sensing amplification strategy, with particular attention to the way polymerase-driven strand displacement is translated into programmable analytical signals. The review moves from the molecular design of SDA—including enzyme selection, catalytic cascades, CRISPR coupling, interfacial confinement, and microfluidic implementation—to its biomedical, food, and environmental uses. It then turns to the features that matter most for translation: portable formats, regenerative nanomachines, molecular logic, data-assisted interpretation, and complete sample-to-answer operation. Rather than ranking platforms by detection limit alone, the analysis considers matrix tolerance, assay time, reagent stability, contamination control, manufacturability, and workflow completeness as practical measures of translational readiness.

The distinctive contribution of this review lies in connecting four levels that are often considered separately: molecular SDA design, recognition-to-DNA transduction for non-nucleic-acid analytes, sample-dependent analytical constraints, and integrated system implementation. The discussion does not stop at sensitivity. It asks whether an SDA system remains reliable when the sample becomes more complex, the workflow becomes longer, reagents must be stored, and the assay has to be manufactured reproducibly. Intelligent readout and commercialization are considered within the same context. From this perspective, the useful question is not how much amplification can be added, but how much complexity an assay can tolerate before its practical advantage begins to disappear.

## 2. Molecular Design Principles and Analytical Integration of SDA

### 2.1. Enzyme-Assisted Isothermal SDA and Programmable Amplicon Generation

Classical SDA is built around two recurring enzymatic steps: strand-displacing DNA synthesis and sequence-specific nicking [2,23]. Primer extension generates a duplex containing the nickase recognition site; cleavage then creates a fresh 3′-OH terminus that can be extended again. Successive rounds of this process continuously release displaced strands and sustain amplification [2,23]. Further polymerase activity pushes the newly formed strand forward and releases the DNA produced in the preceding round, enabling amplification to proceed continuously at a single temperature [23]. What happens to the released strand is dictated largely by circuit design: it may provide the analytical signal itself, prime another synthesis event, operate as a recyclable trigger, or enter a subsequent reaction module. This functional versatility has established SDA and related strand-displacement polymerization chemistries as useful amplification elements in miniaturized biosensing systems [24,25].

SDA gains much of its analytical versatility from the freedom to design displaced strands independently of the original target sequence. Careful selection of templates, primers, and hairpins can therefore direct amplification toward sequence outputs optimized for the intended sensing reaction [25]. This flexibility is demonstrated by an All-U-Want SDA (AUW-SDA), in which interchangeable primer domains can direct the reaction to a G-rich chain (for G-quadruplex/heme colorimetry) or an activating sequence (for CRISPR/Cas12a fluorescence). The same SDA design can be adapted to different signal outputs while keeping the target recognition sequence unchanged [26]. Early cyclic SDA structures similarly linked target recognition with the generation of repeat products, for example, using fluorescence polarization to detect *Salmonella* [27].

Programmable amplicon generation therefore gives SDA a role beyond simple target replication. By controlling the sequence and function of the displaced products, the same amplification chemistry can be adapted to different analytical outputs without redesigning the initial recognition event [28,29,30,31,32]. These products may subsequently enter catalytic circuits, CRISPR-based modules, or other downstream signal-processing pathways, which are discussed in Section 2.2.

### 2.2. Cascaded Amplification, CRISPR Coupling, and Programmable Molecular Outputs

SDA becomes especially versatile when its products are treated not as the final analytical output but as molecular intermediates that can be directed into a second reaction layer. Under isothermal conditions, polymerase-driven strand displacement converts scarce target-derived inputs into abundant, sequence-defined DNA strands. These products can subsequently act as primers, activators, catalytic triggers, or structural inputs for downstream signal-processing modules. In SDA–CHA and SDA–HCR architectures, for example, the displaced strands generated during enzymatic amplification initiate a secondary hybridization cascade, providing an additional level of signal gain without altering the primary amplification mechanism [12,33,34]. Related designs couple SDA to primer-exchange reactions, in which newly released strands enter a second isothermal amplification stage and increase reporter generation [35]. The analytical benefit therefore arises from division of function: SDA establishes the first layer of molecular amplification, whereas the downstream circuit reshapes or further amplifies the resulting signal.

CRISPR coupling builds on the same molecular architecture while introducing sequence-specific signal conversion. SDA first enriches the target-derived sequence, generating enough activators to engage the CRISPR nuclease, which then amplifies the readout through repeated reporter cleavage. T-junction-mediated SDA has been linked to Cas12a for ECL detection of bacterial 16S rDNA [36], while modular SDA–CRISPR systems have supported fluorescence, lateral-flow, and personal glucose-meter readouts from a common amplification architecture [37]. More complex configurations use tetra-way SDA to generate multiple functional strands, allowing Cas12a activation and electrochemical reporting to be coordinated within the same reaction network [38,39]. Similar approaches have been applied to miRNA, piRNA, bacterial DNA, and viral targets, illustrating how the sequence programmability of SDA can be exploited to regulate CRISPR activation rather than merely increase the nucleic-acid copy number [40,41,42,43,44,45].

The main advantage of these cascaded architectures is that signal amplification can be decoupled from the molecular processing that follows. SDA supplies the primary enzymatic gain, whereas CHA, HCR, primer-exchange, CRISPR cleavage, and related modules determine how the amplified information is filtered, converted, or transmitted to the final transducer. This distinction is important because not every strand-displacement event within a multistage assay constitutes SDA. The defining step remains polymerase-mediated synthesis accompanied by displacement and reaction regeneration; subsequent hybridization, cleavage, or structural reconfiguration serves as signal processing. Viewed in this way, SDA is best understood as a programmable molecular source that generates defined nucleic-acid outputs for downstream analytical functions. What changes from classical SDA is not the underlying strand-displacement chemistry, but the fate of the displaced product—where it is directed and what molecular role it takes on once amplification has occurred. More elaborate systems can use these outputs to drive regenerative motion, spatially organized DNA machinery, or autonomous molecular operations; those platform-level developments are considered separately in Section 5.2.

### 2.3. Interfacial Confinement and Spatial Control of SDA

Spatial confinement reshapes not only assay dimensions but also the local reaction environment governing SDA performance. By restricting where amplification occurs and how newly generated strands interact with a sensing interface, electrodes, nanochannels, hydrogels, and porous matrices can regulate local product concentration, mass transport, and signal conversion. Tetrahedral DNA nanotags localized strand-displacement polymerization at the electrode surface, where miRNA recycling proceeded alongside the delivery of redox-labeled DNA nanostructures to the transducer [46]. This spatial strategy has also been applied to solid-state nanochannels. A Klenow/BbvCI-assisted SDA method was used to prepare single-stranded DNA, which was then assembled into a negatively charged nanonet on anodic aluminum oxide [47]. The resulting interfacial charge alters the way ions are transported in the channel, converting molecular amplification into a measurable change in conductivity.

Confinement can also convert SDA products into changes in material properties or fluid transport. Yan et al. used target-initiated strand-displacement polymerization to generate DNA strands that promoted hydrogel assembly; the resulting increase in viscosity altered seepage distance along a paper strip, enabling visual miRNA quantification [48]. In another format, target-triggered SDA was coupled to Cas12a-controlled hydrogel digestion and translated molecular amplification into a distance signal along a porous thread [49]. These systems are mechanistically distinct from stand-alone strand-displacement circuits: amplification is driven by polymerase-mediated DNA synthesis, whereas the confined material provides the physical readout.

These architectures retain the basic nicking–polymerization logic of SDA but intervene at different points in the reaction. Design changes may alter the role of the displaced strand, direct it into a secondary molecular reaction, or localize its production near the sensing interface. Structure-gated architectures act earlier by controlling when amplification begins. The resulting benefits depend on where this intervention occurs, ranging from more flexible readout and greater signal gain to sequence-selective CRISPR processing, improved local transduction, and tighter control over premature reactions. Table 2 compares these functional gains with the limitations introduced by each design.

Spatial organization adds another consideration to SDA: the site of amplification can determine where products accumulate and how they reach the sensing interface. Some use magnetic enrichment or electrophoretic separation to control where SDA products accumulate before detection. SERS-based chip formats, for example, combine nickase/polymerase-driven amplification with magnetic capture and secondary nucleic-acid processing to concentrate amplified products near the Raman-active interface [50]. Microchip electrophoresis has similarly been coupled to SDA for exosome analysis, foodborne-pathogen detection, and multiplex miRNA measurement [51,52]. Structure-gated amplification adds another layer of spatial and kinetic control by limiting premature product formation until the required conformational change occurs [53]. Taken together, these studies indicate that interfacial confinement does more than reduce assay dimensions. It influences where amplification products form, how they move and accumulate, and how efficiently they are converted into a measurable physical signal. Once SDA is brought onto a sensing surface, the observed response reflects both the amplification chemistry and the local transport and interaction of products at the transducer. Portable device implementation and field-oriented readout are discussed separately in Section 5.1.

### 2.4. Recognition-to-DNA Transduction for Non-Nucleic-Acid Analytes

Nucleic-acid targets can engage SDA directly through sequence-specific pairing with primers, templates, or recognition probes. Proteins, small molecules, and other non-nucleic-acid analytes cannot be amplified in this way and must first be translated into a DNA-based trigger [54]. In these assays, target recognition first changes the state of a DNA-containing recognition complex; the resulting structural, spatial, or catalytic event then exposes, releases, assembles, or generates a DNA trigger that can initiate SDA. The recognition reaction therefore determines analyte selectivity, whereas SDA amplifies the nucleic-acid information produced by that recognition event.

Several recurring architectures can be distinguished. Aptamer binding may induce a conformational change or competitive displacement that releases an initiator strand, a strategy applicable to proteins and small molecules. Antibody- or aptamer-mediated proximity recognition can instead bring DNA labels into a productive configuration, allowing target binding to create an amplifiable complex [55]. Enzyme activity may be converted through substrate cleavage or sequence release, while metal ions or other chemical species can activate DNAzymes that expose an SDA-compatible strand [56]. The recognition step varies with the analyte, whereas the downstream route is largely conserved: a DNA signal is generated, amplified by SDA, and passed to the detection stage. This reaction logic remains the same whether the assay is used in clinical testing, food analysis, or environmental monitoring. Application-specific sections therefore emphasize matrix handling, validation, and practical use, whereas the recognition-to-DNA conversion mechanisms are treated here as common analytical design principles [5,6].

The most sensitive point in the assay is the transition from target recognition to amplification. Premature trigger release can start SDA without the analyte, whereas inefficient conversion leaves too little DNA available for amplification. Binding affinity, reaction kinetics, steric accessibility, trigger stoichiometry, and compatibility between recognition chemistry and polymerase/nickase conditions therefore influence assay performance as strongly as the amplification reaction itself. Effective non-nucleic-acid SDA sensing depends on suppressing spontaneous trigger generation while preserving efficient target-induced conversion.

## 3. Biomedical Applications of SDA-Enabled Sensing

### 3.1. SDA-Enabled Sensing of RNA Biomarkers

MicroRNAs are particularly suitable targets for SDA because low abundance, short sequence length, and close homology among family members make direct analysis difficult [57,58,59,60,61]. Polymerase-driven strand displacement provides molecular gain without thermal cycling, while the displaced products can be directed toward fluorescence, electrochemical, CRISPR, or nanostructure-based readouts [62,63,64,65,66,67]. For RNA samples, however, amplification efficiency depends on more than target abundance. Secondary structure, nuclease exposure, sequence similarity, and accessibility of the primer-binding region can all affect initiation and specificity. Probe design must therefore establish selective recognition and stable primer access before amplification begins. What ultimately separates these platforms is how effectively molecular recognition is translated into a DNA signal that can be amplified and meaningfully read out.

**Figure 2:**
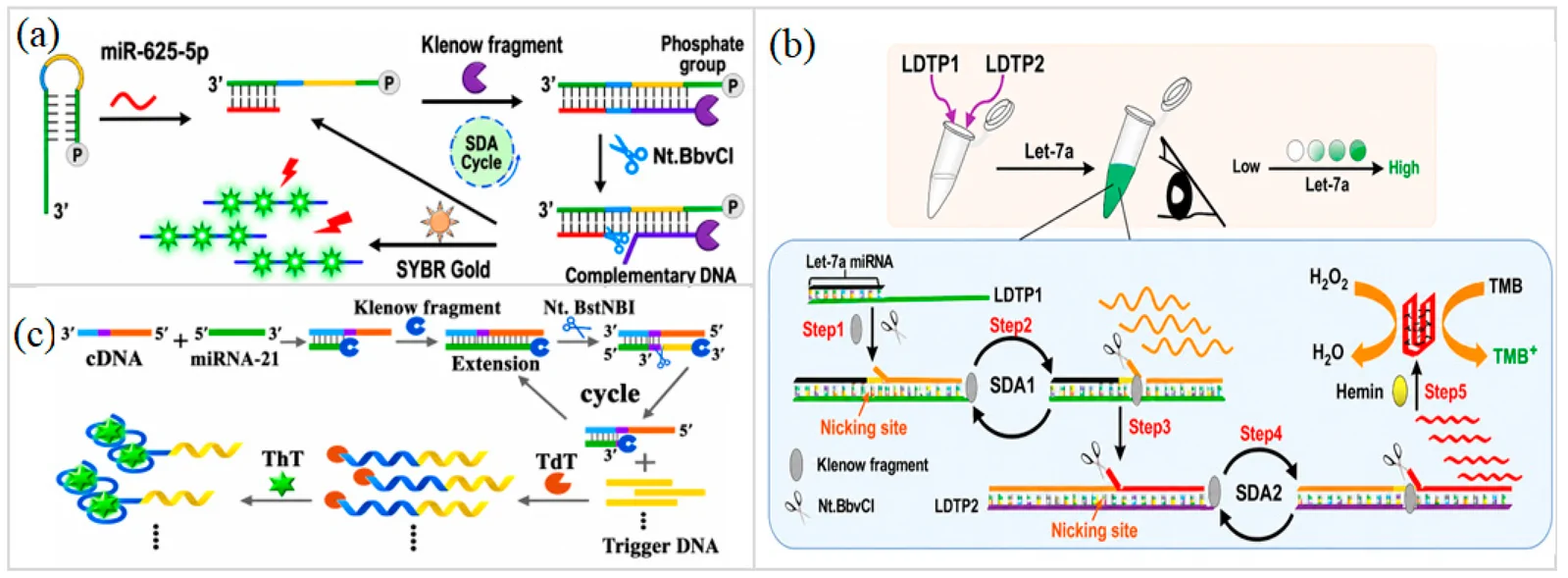
(**a**) Schematic of miR-625-5p detection using hairpin-initiated SDA using Klenow polymerase and Nt.BbvCI, followed by SYBR Gold fluorescence readout. (Reprinted from Ref. [57]). (**b**) Schematic of a Let-7a colorimetric assay using LDTP1/LDTP2-triggered tandem SDA and G-quadruplex/hemin-catalyzed TMB oxidation for visual quantification of miRNA levels. (Reprinted from Ref. [65]). (**c**) Schematic of miRNA-21 detection integrating strand-displacement amplification, TdT-assisted poly(G) extension, and G-quadruplex/ThT fluorescence for sensitive label-free signal amplification. (Reprinted from Ref. [72]).

Early designs relied on comparatively direct fluorescence reporting. Chen et al. [57] coupled polymerase–nicking enzyme cycling to SYBR Gold detection of miR-625-5p (Figure 2a), illustrating a straightforward route in which SDA products are read without an additional signal-processing layer. Such formats reduce molecular circuitry, but fluorescence from complex biological samples can be affected by background nucleic acids, nonspecific dye interactions, and matrix-dependent amplification. More elaborate architectures instead use the displaced strand as a functional intermediate. Xu et al. [65] used a two-stage SDA cascade to generate G-quadruplex/hemin DNAzymes for colorimetric readout (Figure 2b). The amplified RNA signal can ultimately be read by eye without repeated temperature cycling. Adding further reaction steps, however, leaves more room for background leakage and makes both assay timing and color development harder to control consistently.

The analytical reach of SDA has broadened beyond the extensively studied cancer biomarker miRNA-21 [68,69,70,71]. Yan et al. [72] used SDA to convert miRNA-21 recognition into a trigger strand, followed by terminal deoxynucleotidyl transferase extension to generate G-rich products for label-free G-quadruplex/ThT fluorescence, as shown in Figure 2c. The methodological value lies in separating target recognition, enzymatic amplification, and reporter formation into programmable modules. In cell lysates, serum, or other minimally processed specimens, however, each additional module introduces opportunities for nuclease degradation, enzymatic inhibition, nonspecific amplification, and accumulated handling error. A useful feature of SDA is its ability to transform scarce RNA into programmable DNA products without thermal cycling. For clinical assays, the remaining challenge is to make this process faster and easier to implement while retaining internal controls and reliable amplification in minimally processed specimens.

### 3.2. SDA-Enabled Liquid-Biopsy Analysis of Extracellular Vesicles and Circulating Biomarkers

Liquid biopsy is a particularly demanding setting for isothermal amplification, as circulating biomarkers are typically scarce and must be distinguished within the chemically complex background of plasma or serum [73,74,75,76,77,78]. In SDA-based sensing, polymerase extension coupled with sequence-specific nicking or cleavage converts low-copy targets into defined amplification products, distinguishing SDA from enzyme-free strand-displacement circuits [79,80,81,82,83,84,85]. SDA–HCR and microchip electrophoresis platforms further show that this chemistry can be integrated with exosome capture and vesicle-associated biomarker analysis [34,51,86,87].

**Figure 3:**
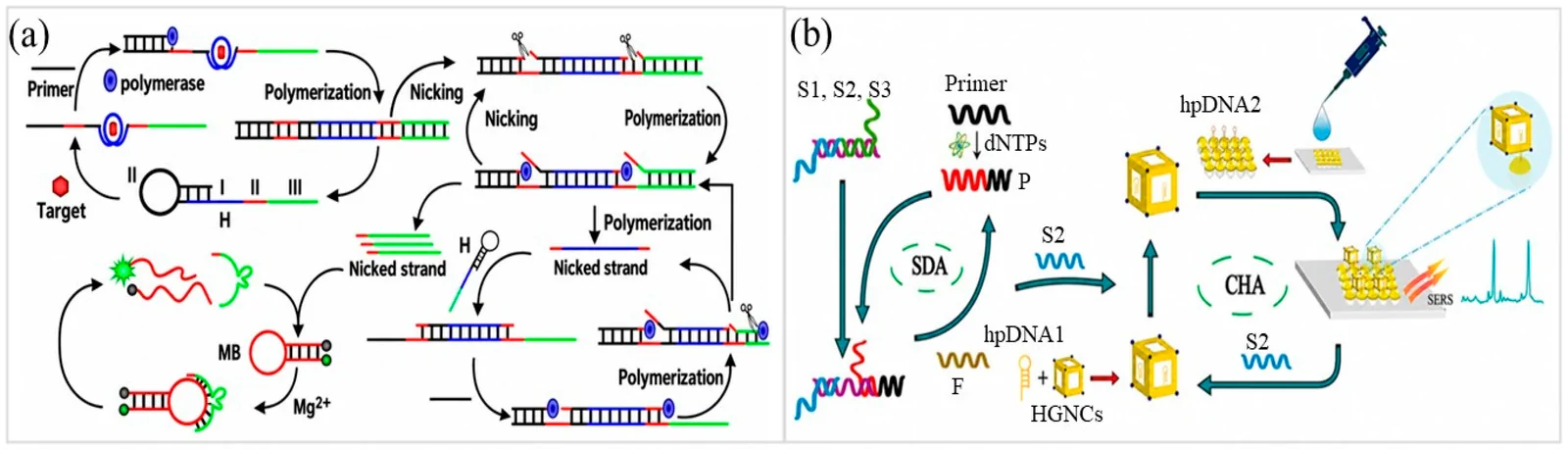
(**a**) Schematic of CEA-triggered isothermal exponential amplification, integrating polymerization–nicking cycles with Mg^2+^-dependent DNAzyme cleavage of molecular beacons for amplified fluorescence detection. (Reprinted from Ref. [77]). (**b**) Schematic of an SDA–CHA dual-amplification SERS biosensor for ultrasensitive telomerase activity detection using Au–Ag nanocage probes and an Au@SiO_2_ array substrate. (Reprinted from Ref. [84]).

SDA enters the extracellular-vesicle workflow relatively late. The sample has usually undergone vesicle isolation, membrane disruption, and RNA recovery before it reaches the amplification step, and variability introduced along this path can carry through to the final readout. The exosomal miR-21 assay of Xia et al. [75] makes this trade-off clear: high amplification sensitivity was achieved, but only after ultracentrifugation, RNA extraction, and multistep electrode preparation. Protein assays face another barrier because protein binding must first be converted into a nucleic-acid event. For carcinoembryonic antigen (CEA), Wei et al. [77] accomplished this with an aptamer-derived trigger for SDA (Figure 3a). The added conversion step makes protein sensing possible, but serum processing, recognition efficiency, and amplification time remain part of the overall assay burden.

Circulating tumor DNA extends SDA beyond expression analysis toward sequence-variant discrimination. Ma et al. [79] combined enzyme-assisted SDA with high-performance liquid chromatography (HPLC)–fluorescence to resolve wild-type and mutant BRAF V600E, EGFR T790M, and KRAS 134A ctDNA in serum. SDA converted each target into separable G-quadruplex probes while preserving single-nucleotide discrimination. In ctDNA analysis, a rare mutant sequence must be distinguished from an overwhelming excess of nearly identical wild-type DNA. Selectivity must therefore be established before SDA proceeds, or built into its initiation step, to prevent background templates from being amplified along with the target variant.

SDA can also broaden liquid-biopsy analysis beyond nucleic-acid abundance. Shen et al. [84] coupled SDA–CHA with telomerase activity in peripheral-blood mononuclear-cell lysates, converting the enzymatic signal into SERS hot spots formed at Au–Ag nanocage/Au@SiO_2_ interfaces, as shown in Figure 3b. Enzyme-activity measurements in cell lysates place a different burden on the assay because the analyte is a catalytic function rather than a molecular concentration alone. Sample preparation must therefore preserve enzymatic activity while limiting interference from endogenous proteins, nucleases, and other lysate components. Cascaded SDA–CHA and SERS amplification can provide substantial analytical gain, but reproducibility increasingly depends on reaction timing, probe assembly, and formation of consistent plasmonic hot spots. Across these studies, SDA adapts readily to diverse targets and sensing formats, but long amplification times, pre-analytical processing, and elaborate interface preparation still slow its progress toward rapid liquid-biopsy analysis. Table 3 collects the experimental details reported for selected SDA assays targeting circulating biomarkers, including sensing elements, detection formats, working ranges, and detection limits (LODs). Reaction temperature and duration vary from study to study and are presented only to document how each assay was performed; they should not be interpreted as standard conditions for SDA.

**Table 3:** Reported analytical characteristics of representative SDA-based assays for RNA biomarkers, extracellular vesicles, and circulating biomarkers.

Detection Target	Detection Mode	Active Substance/Sensing Element	Reported SDA Condition/Time	Reported Detection Range and LOD	Ref.
Tumor exosomes (CD63)	DPV	CD63 aptamer, MBs, KF, Nt.BbvCI, HRP/Au electrode	SDA: 37 °C, 40 min; HCR: 37 °C, 15 min	R: 44–8.8 × 10^6^ particles/μL, LOD 18 particles/μL	[34]
miR-222	FL	DNAzyme 1/2–substrate system; Nb.BbvCI-mediated SDA; CRISPR/Cas12a–crRNA	SDA: 25 °C, 60 min; t Cas12a: 37 °C, 40 min	R: 0.1 pM–1 nM, LOD: 33.5 fM	[58]
miRNA-373	Colorimetry	TSC, dumbbell template, phi29 DNA polymerase	SDA–TIRCA system, optimum 0.5 U/μL	R: 50 fM–500 nM, LOD: 33.4 fM	[59]
miRNA-21	FL	RRO recognition probe; Bst/Nb.BsmI SDA	SDA: 56 °C, 30 min; one-pot SDA–hHCR	R: 100 fM–100 pM, LOD: 82 fM	[60]
miRNA-21	FL	SDA template; Bst DNA polymerase; Cas12a–gRNA + fluorescent reporter	SDA: 55 °C, 30 min; Cas12a trans-cleavage at 37 °C for 30 min	R: 0.05–25 pM and 25–500 pM, LOD 10.1 fM	[63]
miRNA-21	Visual/colorimetric (UV–vis)	SDA-generated DNA activator; CRISPR/Cas12a–crRNA	SDA: 55 °C, 30 min; Cas12a: 37 °C, 40 min	R: 1 pM–1 nM, LOD: 420 fM	[66]
miRNA-155	FL	Nt.BbvCI-mediated SDA; Cas12a–crRNA + FQ reporter	SDA: 37 °C, 90 min; Cas12a: 60 min	R:1 pM–10 nM, LOD 0.166 pM	[69]
miR-31	ECL, signal-on	Klenow fragment; CRISPR/Cas12a; DNA1-Fc	ISDA: 37 °C, 30 min; Cas12a reaction: 37 °C, 40 min	R: 10 aM–100 pM, LOD 1.67 aM	[70]
Exosomal miR-15/-21/-145/-155/-423/-451	Terahertz	SDA-generated trigger DNA; biotin probes	SDA: 37 °C, 2 h; HCR: 37 °C, 2 h	R: 1 fM–100 pM, LOD 12.54–26.59 aM	[76]
ctDNA	PEC	Self-priming HP template; S1/S2 SDA products	MC-SDA: 37 °C, 2 h,	R: 0.1 fM–100 pM, LOD: 21.8 aM	[80]
ctDNA	FL	DSH/G4–ThT	SDA: 37 °C, 120 min	R: 100 fM–100 nM, LOD 19.1 aM	[81]
sPD-L1/exoPD-L1	Colorimetric/UV–Vis	Nb.BbvCI, phi29 DNAP, Fe-MOF/Fe_3_O_4_, TMB	E-SDA: 33 °C, 60 min; assembly: 30 min	R: 5–10^4^ pg/mL, LOD 5 pg/mL	[82]
sEVs	Colorimetric	CD63 aptamer/SMBs/DNAzyme	SDA: 37 °C, 60 min	R: 10^3^–10^7^ particles/μL, LOD 267 particles/μL	[86]

E-SDA: enzyme-powered SDA; Fc: ferrocene; FL: fluorescence; hHCR: high-temperature hybridization chain reaction; ISDA: isothermal strand-displacement amplification; KF: Klenow fragment; MB: magnetic bead; LOD: detection of limit; PEC: photoelectrochemical; RC-SDA: rolled-circular SDA; sEVs: small extracellular vesicles; TIRCA; toehold-initiated rolling circle amplification.

### 3.3. SDA-Enabled Genotyping and Epigenetic Analysis

Genetic-variant analysis places a different demand on SDA than abundance-based biomarker sensing [88,89,90,91,92]. Amplification alone is insufficient; sequence discrimination must be established before molecular multiplication begins [93,94,95]. Accordingly, recent SDA-based genotyping platforms place a selective enzymatic gate—such as ligation, restriction-endonuclease cleavage, or CRISPR recognition—upstream of polymerase- and nicking-enzyme-mediated amplification [96,97,98]. This ordering is essential because a rare mutant allele may coexist with a large excess of nearly identical wild-type DNA. Once nonspecific amplification begins, downstream signal gain cannot recover the lost sequence selectivity. For genomic DNA, assay performance therefore depends on the fidelity of the recognition gate, accessibility of the target locus, fragmentation state, and the mutant-to-wild-type ratio before SDA is initiated. This arrangement separates mutation recognition from signal gain and avoids reliance on toehold-mediated branch-migration kinetics [99,100].

**Figure 4:**
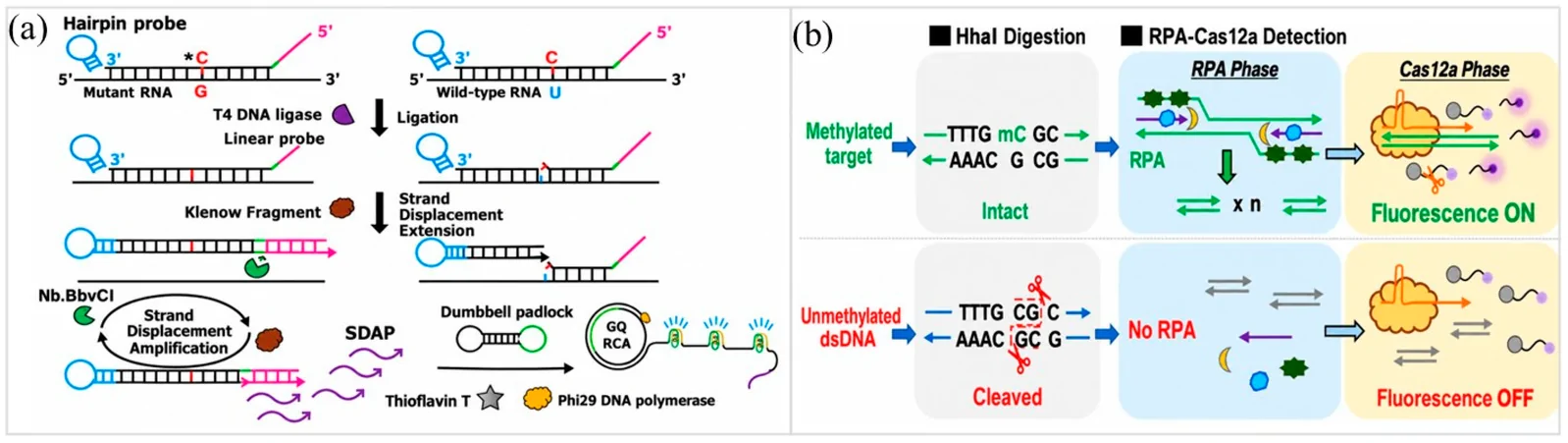
(**a**) Schematic of ligation-assisted tandem SDA–GQ-RCA for selective fluorometric detection of the SARS-CoV-2 L452R single-nucleotide variant using Thioflavin T fluorescence. (Reprinted from Ref. [90]). (**b**) Schematic of HhaI-assisted glycerol-enhanced SDA/RPA–CRISPR/Cas12a detection of DNA methylation, where methylated targets remain intact for amplification and Cas12a fluorescence, whereas unmethylated DNA is cleaved. (Reprinted from Ref. [99]).

In Zhou et al. [89], RC-SDA amplified the EGFR exon-19 deletion signal and generated the DNA required for Cas12a activation, with fluorescence and DPV providing independent readouts. What matters most in this design is the order of events: mutation discrimination comes first, followed by high-gain amplification. For genomic DNA targets separated from the wild-type by only a subtle sequence difference, even limited cross-reactivity at the recognition stage can propagate through SDA and become disproportionately represented in the final signal. Orthogonal readouts can therefore strengthen analytical confidence, but they do not compensate for poor allele discrimination at the input stage. Kyung et al. [90] similarly paired mutation-selective ligation with SDA and G-quadruplex-producing rolling circle amplification (RCA) for single-nucleotide discrimination, as shown in Figure 4a. Synthetic oligonucleotides provide a useful test of sequence fidelity, but full-length viral RNA introduces secondary structure, degradation, reverse-transcription variability, and heterogeneous clinical backgrounds. Translation therefore requires confirmation in intact viral or clinical specimens rather than sequence standards alone.

Methylation itself is not directly amplified; it must first be translated into a sequence-selective trigger for SDA. In the assay reported by Ding et al. [96], GlaI provided this recognition for methylated Septin9, while SDA coupled with CRISPR/Cas12a converted the event into a fluorescent signal. With clinical DNA, methylation heterogeneity and variable DNA quality or enzymatic cleavage can directly alter the template available for SDA. Bisulfite-free schemes reduce DNA damage and processing steps, but methylated and unmethylated sequences must still be distinguished reliably. RC-SDA and GlaI-assisted approaches provide related routes for generating SDA triggers [97,98]. Lu et al. [99] used HhaI digestion to resolve locus-specific methylation associated with nasopharyngeal carcinoma before glycerol-enhanced RPA and CRISPR/Cas12a detection, as shown in Figure 4b. HhaI preferentially cleaved unmethylated DNA, whereas the methylated templates remained intact for subsequent amplification and fluorescent readout. The recognition logic is relevant to SDA-based methylation assays, but the amplification step itself is RPA; the study therefore serves as a mechanistic comparator rather than an example of SDA. Its inclusion is useful because it highlights the same analytical principle: selectivity is established before amplification rather than created by amplification itself. Across genotyping and epigenetic analysis, the decisive challenge is therefore not greater signal gain but preservation of allele or methylation specificity in heterogeneous clinical DNA. Further progress will depend on accurate allele-fraction measurement, suppression of background amplification, tolerance to complex matrices, and validation with minimally processed clinical samples.

### 3.4. SDA-Enabled Sensing of Biomedical Proteins, Enzyme Activities, and Endogenous Small Molecules

**Figure 5:**
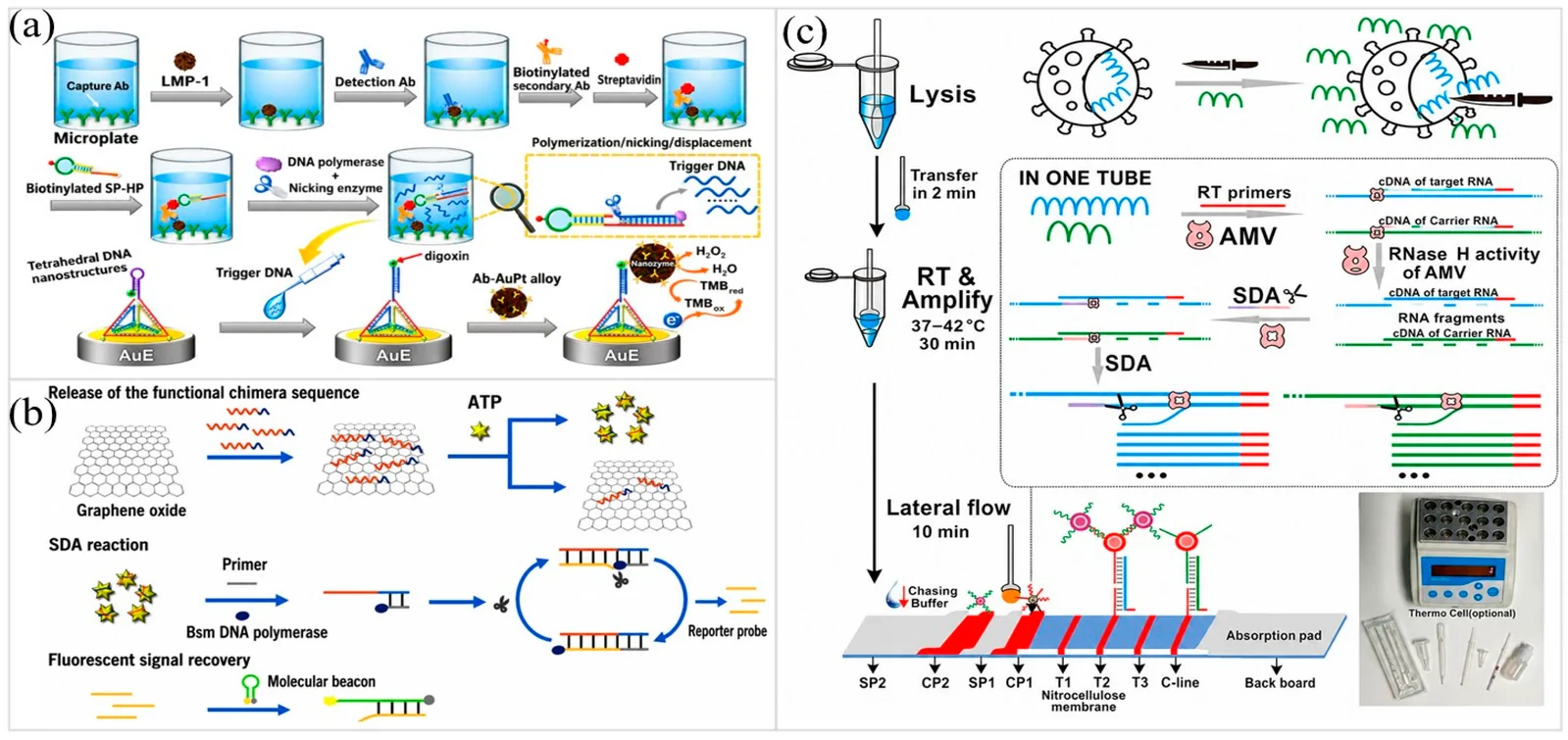
(**a**) Schematic of SDA-mediated nanozyme capture on DNA tetrahedra for ultrasensitive amperometric detection of Epstein–Barr virus latent membrane protein 1, integrating immunorecognition, trigger-DNA amplification, and AuPt nanozyme catalysis. (Reprinted from Ref. [102]). (**b**) Schematic of an ATP-specific fluorescent aptasensor integrating graphene oxide-mediated chimera release, Bsm DNA polymerase/Nb.bpu10I-driven SDA, and molecular-beacon fluorescence recovery. (Reprinted from Ref. [111]). (**c**) Workflow of an extraction-free multiplex viral RNA assay integrating rapid lysis, one-step reverse transcription–strand-displacement amplification, and lateral-flow detection of influenza A, influenza B, and SARS-CoV-2. (Reprinted from Ref. [117]).

The recognition-to-DNA conversion described in Section 2.4 also underpins SDA assays for proteins, enzyme activity, and endogenous small molecules. Aptamers, antibodies, and enzyme-responsive probes first convert target binding or catalysis into a DNA trigger, which then enters the SDA reaction [101,102,103,104]. Reliable detection depends on more than target binding alone. Recognition must remain selective in the biological matrix, and the resulting event must produce a nucleic-acid trigger with sufficient consistency to support downstream amplification. Wang et al. [104] used an aptamer-mediated conversion scheme for prostate-specific antigen (PSA), whereas Liao et al. [102] generated the SDA trigger through sandwich immunorecognition of latent membrane protein-1 (LMP-1) (Figure 5a). These approaches illustrate the same design principle: molecular recognition determines whether amplification begins, whereas SDA supplies the nucleic-acid gain required for downstream optical or electrochemical transduction. In serum-based assays, improving trigger fidelity and reducing matrix-dependent binding errors may therefore be more important than further lowering the nominal LOD.

Enzyme activity can be linked to SDA through the same general strategy, except that the DNA trigger is produced by catalysis rather than released upon target binding [105,106,107,108,109]. Sample preparation becomes particularly important when the assay measures enzyme activity rather than target abundance. Nuclear extracts, cell lysates, and serum may carry inhibitors, nucleases, salts, and competing proteins that interfere with the catalytic step and continue to affect the downstream SDA reaction. Cui et al. [106] linked FEN1 cleavage to SDA and a subsequent rolling-circle amplification step, creating a cascade for enzyme-activity measurement and inhibitor screening. Its performance, however, rests on maintaining FEN1 activity, suppressing nonspecific cleavage, and ensuring that downstream amplification remains tied to the original catalytic event. The extended multistep workflow also limits practical implementation.

Small molecules present an additional challenge because they contain no inherent nucleic-acid sequence for direct amplification. Aptamer-mediated SDA circumvents this limitation by translating ligand-induced conformational changes into accessible primer or trigger domains [110,111,112,113,114,115]. In this setting, analytical specificity depends heavily on aptamer affinity, conformational switching efficiency, and discrimination against structurally related compounds before SDA begins [110,113]. Chen et al. [111] used ATP-induced reorganization of an aptamer/graphene-oxide probe to release the SDA initiator (Figure 5b), while related allosteric designs couple the resulting products to CRISPR-based signal conversion [112]. Serum proteins, ionic strength, and competing ligands can perturb these equilibria, so matrix robustness must be established independently of amplification gain. Across proteins, enzyme activities, and small molecules, the critical challenge lies less in amplification efficiency than in converting molecular recognition into a precise and reproducible SDA trigger.

### 3.5. SDA-Enabled Viral Diagnostics

Viral diagnostics place a different set of demands on SDA. Clinical utility depends not only on sensitivity but also on rapid sequence recognition, tolerance to respiratory or blood-derived matrices, minimal sample preparation, and compatibility with simple readout formats [116,117,118,119,120,121]. Viral RNA is particularly vulnerable to degradation, whereas swab eluates, saliva, mucus, blood, and cell lysates may carry inhibitors that affect reverse transcription, polymerase activity, or nicking efficiency. The analytical workflow must therefore balance rapid lysis with preservation of target nucleic acid and compatibility with downstream amplification. SDA has been combined with reverse transcription for RNA targets, CRISPR for sequence confirmation, and lateral-flow strips for visual detection. This modular design supports viral RNA, DNA, and protein-marker analysis without relying on repeated PCR temperature cycling.

Lu et al. [117] integrated reverse transcription with single-direction SDA in a one-tube format followed by AuNP lateral-flow visualization (Figure 5c). The methodological value of this design lies in minimizing transfer steps between lysis, reverse transcription, amplification, and readout. Extraction-free testing reduces equipment and operator burden, but it also exposes SDA directly to salts, proteins, nucleases, and other components of nasal-swab matrices. Viral sensing can also combine nucleic-acid and protein markers within a common amplification framework. In Zhang et al. [119], proximity recognition linked HIV-1 gag DNA and p24 to an SDA–CRISPR/Cas12a readout. Multimarker analysis can improve diagnostic confidence because nucleic-acid and protein signals describe different aspects of infection, but combining them also introduces unequal target abundance, different sample-preparation requirements, and additional recognition steps. A common amplification architecture is therefore most useful when each input remains independently traceable and does not introduce cross-interference into the other channel. Such multimarker formats can strengthen diagnostic confidence by linking orthogonal viral signatures to the same analytical platform, although their added molecular complexity must be balanced against assay time and operational simplicity.

SDA-based viral testing reduces dependence on thermal cycling, but lowering the LOD further will do little to overcome the practical barriers that still limit translation. Greater value will come from shorter reaction sequences, simpler sample conditioning, stabilized reagents, closed handling, and validation across larger and more heterogeneous clinical cohorts. A sensitive assay may still perform poorly with respiratory or blood samples if lysis is inefficient, matrix effects are strong, or reagents deteriorate during storage. Meaningful benchmarking should therefore include these factors, together with invalid-test frequency, operator time, and concordance with established molecular diagnostics under realistic sample conditions. Table 4 compiles the reported analytical characteristics of representative SDA-based assays for genotyping, epigenetic analysis, proteins, enzyme activities, small molecules, and viral targets. The experimental conditions shown in the table reflect the settings adopted in the cited studies and are included for comparison rather than as general methodological recommendations.

## 4. Food and Environmental Applications

### 4.1. SDA-Enabled Food Safety Analysis: Contaminants, Pathogens, and Authenticity

Food applications do not require a different SDA mechanism. They use the same recognition-to-DNA conversion described in Section 2.4 but place it in a more demanding analytical environment. Matrix interference, target extraction, and regulatory thresholds become the dominant concerns, shifting attention from amplification chemistry to sample handling and assay validation. In small-molecule assays, aptamer binding can uncover or liberate the strand that initiates SDA. Pathogen assays use a broader set of entry points, ranging from direct recognition of genomic DNA to affinity-based schemes that translate intact-cell binding into a DNA trigger.

**Figure 6:**
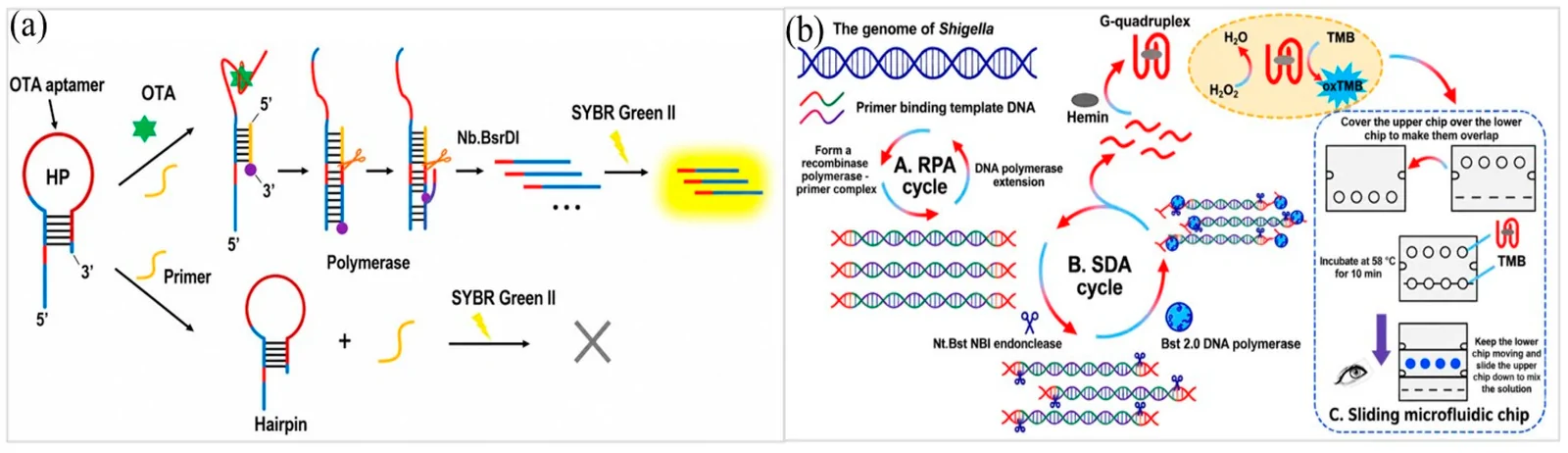
(**a**) Schematic of an OTA aptamer-triggered hairpin SDA assay, where ochratoxin A initiates polymerase/nicking amplification and SYBR Green II fluorescence, while no target yields no signal. (Reprinted from Ref. [123]). (**b**) Schematic of cascaded RPA–SDA for *Shigella ipaH* detection on a sliding microfluidic chip, using G-quadruplex/hemin DNAzyme-catalyzed TMB oxidation for visual colorimetric readout. (Reprinted from Ref. [134]).

In food-contaminant assays, SDA is typically introduced after target recognition to build the signal isothermally; reported applications include mycotoxins, antibiotics, and pesticides measured by fluorescence, electrochemical, photothermal, and ECL readouts [122,123,124,125,126,127,128,129]. For small-molecule contaminants, recognition alone is not enough; the binding event must produce DNA that can enter SDA. Patulin detection in apple juice illustrates this principle, with aptamer-mediated trigger release followed by amplification and optical or strip readout [125]. Chloramphenicol assays instead use the amplified strands to power DNA-walker electrochemiluminescence [128], whereas carbaryl schemes route them through CHA and G-quadruplex–hemin signaling [129]. Their practical performance is often governed by matrix interference, target recovery, separation steps, and overall assay length. A specific study, such as Guo et al. [123], established an early food-safety application by coupling aptamer recognition with polymerase/nickase-driven SDA for ochratoxin A (OTA), as shown in Figure 6a. Real-time SYBR Green II fluorescence quantified OTA over 0.01–50 ng/mL, with an LOD of 0.01 ng/mL; wheat and oat recoveries reached 96–100.1%. Removing the labeling step makes the assay simpler than antibody-based formats, but fluorescence detection still depends on dedicated optical instrumentation.

Foodborne-pathogen sensing places particular emphasis on target recovery before amplification. Intact bacterial cells must first be isolated from protein-, lipid-, and carbohydrate-rich food matrices or converted into an accessible nucleic-acid input before SDA can proceed reliably [130,131,132,133,134]. Aptamer-assisted magnetic capture provides one route to this conversion, as illustrated by the *E. coli* O157:H7 assay of Pang et al. [132], in which selective cell capture was coupled to Klenow/Nb.BbvCI-mediated SDA and G-quadruplex/ThT fluorescence. The loss of sensitivity in milk relative to pure culture underscores a recurring practical constraint: incomplete enrichment and matrix interference may dominate assay performance before amplification becomes limiting. Zhou et al. [134] first amplified *Shigella* by RPA and then performed SDA on a sliding microfluidic chip, as shown in Figure 6b. Amplification proceeds at 39 °C before a 10 min SDA step at 58 °C, where G-quadruplex/hemin DNAzymes generate a visible chromogenic signal. Testing of spiked lettuce gave a detection limit of 3.5 × 10^−4^ ng/μL. The pump-free design favors portable use, although extraction and RPA are still performed separately.

SDA remains less widely explored for food-authenticity analysis than for pathogen or contaminant detection. In this setting, assay performance depends strongly on the recovery and integrity of species- or transgene-specific nucleic acids from processed food matrices, where DNA degradation, co-extracted inhibitors, and closely related sequences can compromise both recognition and amplification [135,136,137,138,139]. Selective processing before SDA can therefore be particularly valuable. CpAgo-mediated cleavage, for example, can generate a sequence-defined trigger that improves target discrimination before subsequent amplification and signal conversion, as demonstrated in seafood authentication [136]. Coupling SDA products to G-quadruplex/hemin-assisted SERS further expands readout sensitivity, but the practical benefit depends on how many upstream and downstream steps are required. Wu et al. [137] used a hybrid PCR–isothermal strand-displacement scheme to identify genetically modified soybean and processed soy foods. PCR introduced nicking and G-quadruplex motifs, enabling displacement-generated G-rich reporters for NMM IX fluorescence. Genetically modified content spanned 0.5–50%; exponential amplification lowered the LOD from 0.2% to 0.02% and supported detection in soy milk and tofu. The sensitivity gain still depends on upstream PCR, so thermal cycling remains part of the workflow and weakens one of SDA’s main practical advantages: operational simplicity.

Food-safety applications expose different weaknesses in SDA depending on what is being measured. For small-molecule contaminants, the main difficulty often comes before amplification: target binding must be converted into a DNA trigger with little nonspecific release. Recovery from the food matrix, aptamer selectivity, and interference with the recognition step can therefore determine assay reliability as much as the SDA reaction itself. Pathogen analysis places greater emphasis on cell capture, enrichment, nucleic-acid accessibility, and suppression of food-derived inhibitors before amplification begins. Authenticity testing introduces a different constraint, as processing can fragment DNA and reduce the sequence information available for species- or transgene-specific discrimination. Across all three applications, additional amplification cascades and nanomaterial-assisted readouts can improve analytical sensitivity, but they also increase reaction steps, handling burden, and opportunities for variability. Broader translation will therefore depend on matrix-tolerant sample preparation, fully isothermal reaction schemes, simplified signal acquisition, stable reagents, and closed sample-to-answer integration rather than further gains in nominal sensitivity alone.

### 4.2. SDA-Enabled Environmental Sensing: Toxic Metals and Resistance-Gene Monitoring

Environmental sensing shifts the difficulty away from the amplification mechanism itself and toward the sample environment. Ionic interference, matrix composition, enrichment, and field handling can strongly influence the assay. Metal ions must first be translated into a DNA trigger, often through a DNAzyme or related responsive probe; resistance genes, by contrast, can be recognized and amplified directly.

**Figure 7:**
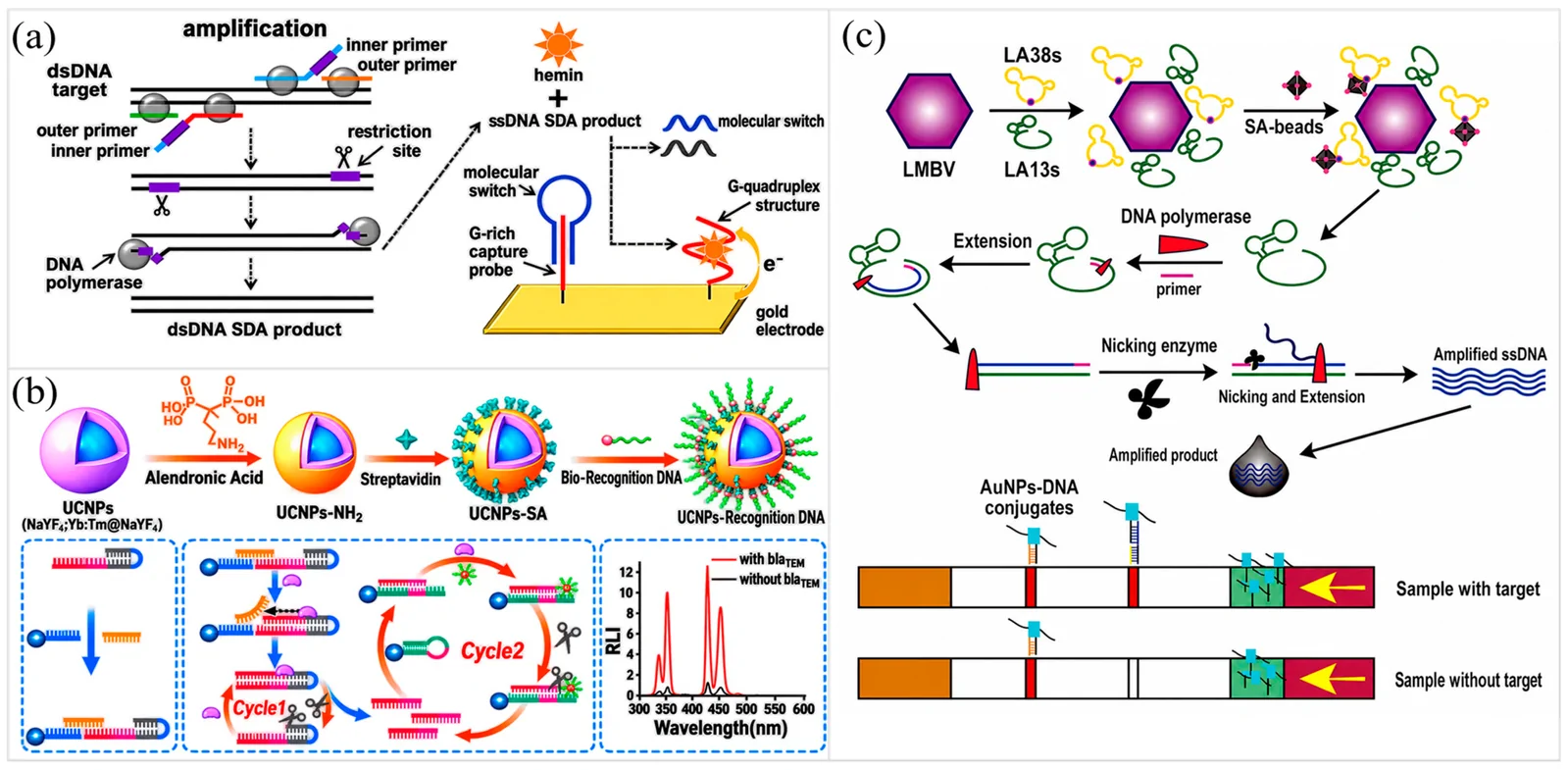
(**a**) Schematic of an electrochemical SDA biosensor in which amplified ssDNA triggers molecular-switch capture on a gold electrode, forming a hemin/G-quadruplex signaling structure for electron-transfer readout. (Reprinted from Ref. [145]). (**b**) Schematic of a UCNP-based dual-cycle SDA nanosensor for ultrasensitive ***bla***_TEM_ detection, integrating magnetic enrichment, polymerase–nicking amplification, DNA recognition, and amplified upconversion luminescence. (Reprinted from Ref. [148]). (**c**) Schematic of an aptamer-based SDA lateral-flow biosensor for LMBV detection, integrating magnetic capture, nicking–extension amplification, AuNP–DNA conjugates, and visual test-line readout. (Reprinted from Ref. [118]).

Environmental SDA assays face a different set of methodological constraints because target recognition must remain selective across water samples with widely varying ionic strength, organic content, and potential amplification inhibitors [140,141,142,143,144,145]. For non-nucleic-acid contaminants such as metal ions, the recognition event must first be converted into an SDA-compatible DNA input before amplification can proceed. DNAzyme-mediated cleavage provides one route to this conversion, as illustrated by Ni^2+^ sensing, where target-dependent cleavage initiates polymerase strand-displacement amplification and the resulting DNA products are subsequently enriched for electrochemical readout [143]. In this context, magnetic enrichment can improve target collection and reduce interference before signal transduction, but it also adds handling steps to the assay. Leonardo et al. [145] described SDA as a low-temperature amplification chemistry well suited to miniaturized sensing (Figure 7a). At 37 °C, inner and outer primers work with a nicking endonuclease and strand-displacing polymerase to drive exponential amplification, while aptamer–G-quadruplex/hemin architectures convert the products into an electrochemical signal. Its suitability for compact devices is promising, although background amplification and full device integration remain unresolved.

Environmental antibiotic-resistance-gene (ARG) surveillance places particular emphasis on efficient target recovery and reliable amplification in chemically heterogeneous water matrices. SDA offers an isothermal alternative to PCR by coupling nicking–polymerization with downstream optical or spectroscopic readout, but assay performance remains strongly dependent on enrichment efficiency, inhibitor tolerance, and the ability to distinguish multiple resistance determinants within the same sample [146,147]. SERS and upconversion-based formats provide sensitive signal transduction [148,149,150], although their practical value depends less on nominal detection limits than on how effectively multiplex recognition can be integrated with matrix-tolerant sample preparation. A one-pot cascade nicking–polymerization/SERS strategy has been used for simultaneous bla-TEM and bla-CTX-M-1 analysis [150], illustrating the value of multiplex SDA for broader resistance profiling. Magnetic capture and nanoprobe processing can improve target isolation and signal quality, but these additional steps still complicate true sample-to-answer operation. Zhang et al. [148] retained the same SDA-like nicking–polymerization logic but replaced Raman tags with a near-infrared upconversion nanoparticle (UCNP) reporter (Figure 7b). After magnetic enrichment, two successive 50 min BbvCI reactions were run at 37 °C, bringing the LOD down to 0.093 aM. The results were highly consistent with qPCR in seven environmental samples. NIR readout suppresses autofluorescence and photobleaching, but nanoprobe fabrication remains technically demanding. These studies suggest that SDA is most valuable when paired with selective enrichment and multiplex readout.

### 4.3. Field-Oriented SDA for Decentralized Food and Environmental Analysis

Field-oriented SDA increasingly depends on workflow integration rather than amplification performance in isolation. Reliable operation outside the laboratory requires target capture, sample conditioning, amplification, fluid transfer, and readout to function as a connected sequence with limited manual handling [151,152,153,154,155,156]. Aptamer-based recognition can enrich bacterial targets before SDA, while microchip electrophoresis provides a route for separating and reading the resulting products, as demonstrated for Salmonella Typhimurium [52]. The analytical value of such schemes is clear, but their field utility is constrained when binding, amplification, and instrument-dependent separation remain discrete steps. Zhang et al. [118] developed a more field-oriented SDA format by coupling two LMBV-specific aptamers with magnetic capture and an AuNP lateral-flow readout, as illustrated in Figure 7c. Detection was not confined to purified virus preparations; LMBV was also identified in infected-cell lysates and in liver, spleen, and kidney tissues. The LOD in infected flounder cells is 8 × 10^1^ cells/mL. The workflow avoided nucleic-acid extraction and thermal cycling, making it less cumbersome than PCR; however, the lower number of positive field specimens points to a remaining limitation in practical diagnostic sensitivity. Field translation depends less on extreme LODs than on closed sample preparation, stable reagents, low-power incubation, and unambiguous readout. Reviews [151,152] likewise identify workflow integration and matrix tolerance as the main barriers separating sensitive SDA chemistry from point-of-use analysis.

For SDA to work reliably outside the laboratory, assay time, sample handling, power use, readout, and reagent stability all need to remain manageable. Just as important is the way amplification is integrated with fluid control, contamination prevention, and tolerance to complex sample matrices. Table 5 summarizes representative food, environmental, and field-oriented SDA systems in terms of target, reaction conditions, sensing elements, analytical performance, and practical format.

## 5. Integrated, Automated, and Intelligent SDA Platforms

### 5.1. Portable Microfluidics, Paper Devices, and Self-Powered Readout

**Figure 8:**
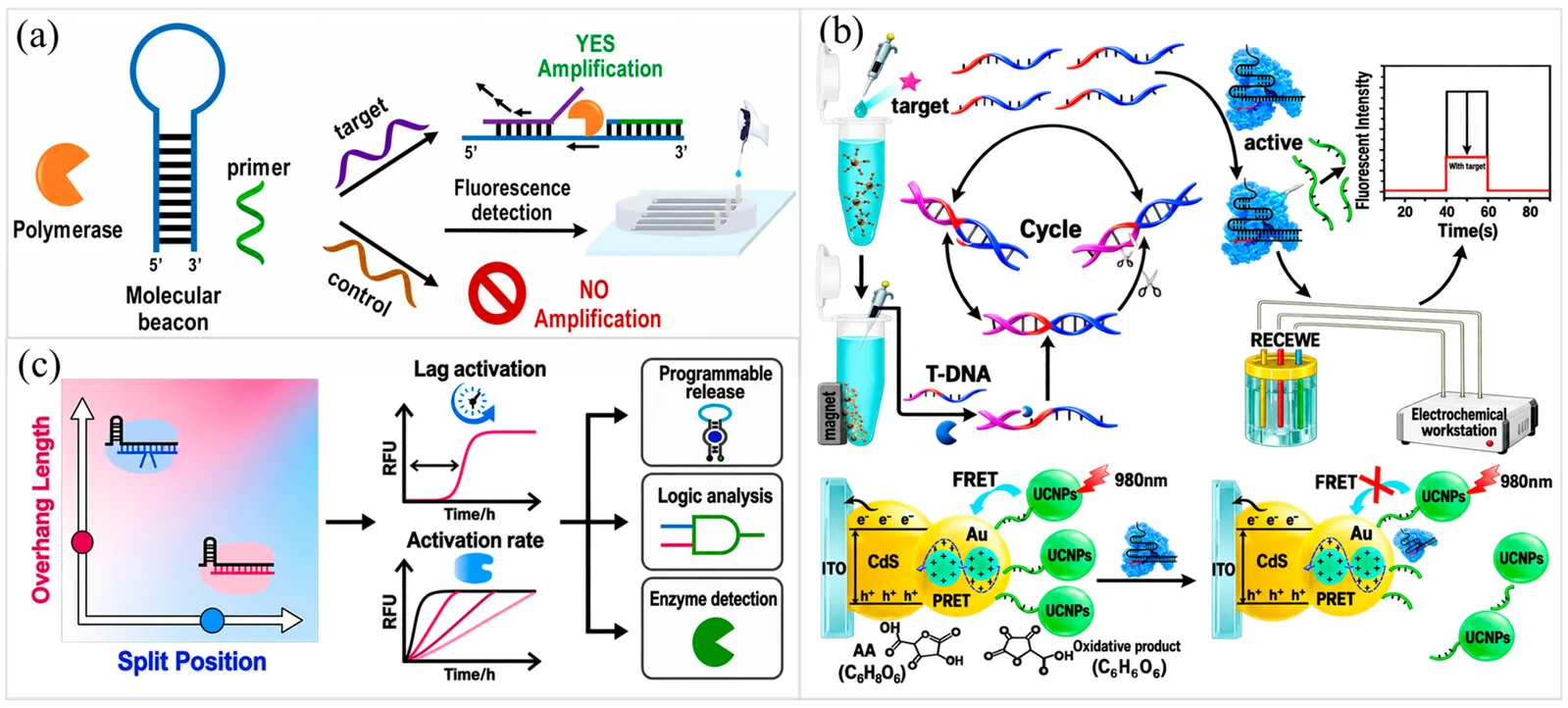
(**a**) Schematic of molecular beacon-mediated microfluidic SDA fluorescence detection, where target-triggered amplification generates a positive fluorescent signal. (Reprinted from Ref. [158]). (**b**) Schematic of an SDA-amplified CRISPR-Cas14a upconversion photoelectrochemical sensor, integrating magnetic target conversion, ssDNA amplification, Cas14a trans-cleavage, and FRET/PRET-regulated photocurrent readout. (Reprinted from Ref. [163]). (**c**) Schematic of SDA-amplified CRISPR-Cas14a upconversion photoelectrochemical sensing, integrating magnetic target recognition, Cas14a trans-cleavage, FRET/PRET modulation, and photocurrent readout. (Reprinted from Ref. [168]).

Portable SDA platforms increasingly couple isothermal amplification with lateral-flow, paper, microfluidic, photothermal, and self-powered electrochemical readouts [18,157]. Recent systems reduce instrumentation and thermal demands, but sample-to-answer translation still depends on faster preparation, reagent stability, closed handling, quantitative readout, and matrix robustness [158,159,160]. For miR-127-5p analysis, Bellassai et al. [158] worked with a 1 μL synovial-fluid sample on a PDMS microfluidic chip, where hairpin-assisted isothermal circular strand-displacement amplification was carried out, as shown in Figure 8a. After a 120 min reaction at 30 °C, SYBR Green fluorescence enabled quantification of miR-127-5p over 0.02–0.50 pM, with the lowest discernible signal corresponding to 34 fM. Avoiding RNA extraction and thermal cycling simplified the assay, although the long amplification time and microscope-based readout still work against true portability.

Paper-based and lateral-flow devices offer a practical route toward decentralized SDA testing. Yan et al. [48] converted miRNA-initiated SDA into DNA-hydrogel formation and a distance-based paper readout, reaching a 1 fM detection limit and showing good agreement with qRT-PCR in human serum. The inexpensive visual format is promising for field use, although reliable quantification still depends on reproducible wicking and well-controlled hydrogel properties. Lu et al. [117] devised an RT-SDA lateral-flow assay (LFA) for influenza A, influenza B, and SARS-CoV-2 using AuNPs as the visual reporter. Viral lysates and spiked nasal-swab specimens could be examined without a separate nucleic-acid purification step; amplification took about 30 min, and the lowest reported detection level was 20 copies/μL. The simplified workflow favors field use, although matrix-dependent sensitivity loss and tight lysis timing remain practical constraints. In sugarcane extracts, Zhang et al. [156] used CRISPR-assisted SDA as the core amplification step in a self-powered scheme for *Sporisorium scitamineum* detection. The amplified signal was converted electrochemically through Fe_3_O_4_@AuNP nanozyme electrodes and further enhanced by capacitive coupling. The platform has an LOD of 41.56 aM and a recovery rate of 92.2–103.5%, demonstrating good analytical performance in the tested matrices. Sensitivity is not the main obstacle to field use. More pressing are reagent stability, simpler on-device fluid handling, shorter assay times, and reliable operation with minimally treated samples.

### 5.2. Regenerative SDA, DNA Nanomachines, and Cell-Free Molecular Automation

Regenerative SDA increasingly functions as a molecular engine rather than a standalone amplifier [161,162]. Coupling cyclic strand generation with DNA walkers, tweezers, spatial nanorobots, CRISPR modules, and self-powered interfaces enables autonomous signal propagation while reducing manual handling across complex analytical matrices [163,164,165]. Regenerative reaction schemes allow SDA to function not simply as a bulk amplifier but as a continuing source of molecular fuel. Dong et al. [32] linked enzymatic SDA to a sterically gated three-dimensional DNA walker for miR-21 sensing. Changing the nanoscale confinement altered the detection limit from 3.6 aM to 35.6 nM and extended the dynamic span from about 10^2^-fold to 1.66 × 10^5^-fold. Cancer-cell and serum data indicate that spatial confinement shifts the balance between sensitivity and working range. The trade-off is tunable, but the multistep protocol still limits rapid routine analysis.

Cheng et al. [64] kept the tripedal DNA walker inactive until target-driven SDA released the strands needed to initiate motion. Electrochemical analysis resolved differences among glioma grades, with miR-375 detectable at 0.83 pM and minimal nonspecific walker activation. The 3 h walking process, however, substantially prolongs the assay and limits its suitability for rapid clinical testing. A further step toward mechanically programmable nanomachines was reported by Yang et al. [113], who coupled aptamer-modified magnetic particles with SDA-driven DNA tweezers for AFB1 and ZEN detection. The LODs were 0.933 and 1.07 pg/mL, respectively. Relative to direct tweezer sensing, SDA improved sensitivity by roughly four orders of magnitude. The ~10^4^-fold sensitivity gain is notable, although magnetic separation and multistep processing still constrain rapid routine screening.

Spatial integration pushes this autonomy further. Wu et al. [155] placed dual-SDA and DNA-walker amplification on a flexible AuNP/sulfur-doped graphyne carbon-cloth platform to detect sugarcane pokkah boeng DNA. The measurable range stretched from 0.1 fM to 10 nM, whereas introducing a second HCR stage brought the LOD down to 16.6 aM. Spatial confinement accelerates amplification, although the layered cascade may complicate routine field deployment. For regenerative molecular automation, Wang et al. [163] integrated aptamer-functionalized magnetic beads with SDA and a UCNP–CdS@Au/ITO photoelectrochemical electrode (Figure 8b). The platform detected T-2 toxin in oat samples at 0.5128 fg/mL and PTK7 in serum at 0.03783 pg/mL. Converting non-nucleic-acid targets into SDA-compatible signals expands the analytical scope of the reaction, but the 3 h amplification period and multistep magnetic/CRISPR processing substantially weaken its suitability for rapid field testing. These designs simplify operation by eliminating thermal cycling and reducing manual handling. Practical automation will be governed less by another drop in nominal LOD than by enzyme compatibility, leakage control, reproducible nanointerfaces, and shorter multistep workflows.

### 5.3. Molecular Logic, Data-Driven Interpretation, and Sample-to-Answer Integration

The emerging value of intelligent SDA lies less in generating stronger signals than in determining how amplified information is interpreted and converted into analytical decisions. Molecular logic, smartphone-based analysis, multimodal signal fusion, and automated readout increasingly extend SDA beyond conventional concentration measurement [166,167]. These approaches differ in complexity, but they share a common objective: to extract more useful information from amplification while reducing dependence on subjective interpretation or laboratory instrumentation [168,169,170].

Molecular logic represents the most direct form of biochemical decision-making. Han et al. [28] constructed gold-electrode interfaces with truncated square-pyramidal DNA nanostructures and paired cyclic enzymatic SDA with ECL for simultaneous analysis of two myocardial miRNAs. Both miRNAs were measured across 10 fM–10 nM. In serum, AND/OR logic combined the two biomarker signals into a single interpretive output rather than reading each target separately. Yin et al. [168] pushed this concept toward programmable pathogen identification (Figure 8c): influenza A/B RNAs were first converted to DNA initiators and amplified by Bst polymerase/Nt.BstNBI SDA at 55 °C for 30 min, after which overhanging Cas12a activators implemented AND, OR, and NOR fluorescence gates. The platform was intended mainly for qualitative classification rather than calibrated quantification. Even so, it illustrates a clear division of roles: SDA expands the molecular signal, while the downstream logic circuit governs its interpretation.

Digital interpretation provides a less chemically complex route to the same goal. Zhang et al. [59] coupled SDA with toehold-initiated RCA in a room-temperature reaction in which proton release produced a visible pH shift. Smartphone RGB analysis translated this optical change into quantitative miRNA measurements over 50 fM–500 nM, with an LOD of 33.4 fM. Eliminating thermal cycling and benchtop optics made signal detection more accessible, but smartphone readout did little to simplify the upstream steps needed to prepare and handle the sample. A related strategy was applied to CD122 sensing by Li et al. [171], who used Phi29/Nb.BbvCI-mediated SDA to generate G-quadruplex nanostructures on AuNP/Zr-MOF/GDY electrodes. Self-powered electrochemical and smartphone-readable colorimetric outputs provided complementary measurements, with LODs of 18.9 and 23.9 aM, respectively. Such dual-mode systems can improve confidence by providing independent signal channels, but multiple readouts do not by themselves constitute intelligent analysis unless the information is subsequently integrated.

Machine learning is used not simply to collect several sensor outputs, but to judge what their combined behavior reveals about the sample. Wen et al. [169] collected electrochemical, colorimetric, and photothermal responses and used algorithmic fusion to extract information from the combined signal pattern rather than from any single channel. This approach enabled internal cross-validation and automated calibration, illustrating how multimodal SDA platforms can extract information from relationships among signals rather than from a single response curve. The benefit, however, depends on the quality and representativeness of the data used for model development. Matrix variation, manufacturing drift, operator-dependent effects, and realistic concentration distributions must be represented adequately; otherwise, algorithmic processing may reinforce assay-specific bias rather than improve analytical reliability [20,22,167,169]. Independent external validation is therefore more important than additional model complexity when such systems are intended for diagnostic or regulatory use.

Data interpretation is only one component of the sample-to-answer operation. A genuinely integrated SDA platform must connect sample preparation, target recognition, amplification, transduction, calibration, and final interpretation with minimal intervention [171,172,173]. Smartphone analysis, molecular logic, self-powered sensing, and machine-learning-assisted fusion can simplify downstream decision-making, but they cannot compensate for unstable reagents, poorly controlled fluid transfer, amplification leakage, or inconsistent sample preparation. The stronger path forward is to design molecular processing, device operation, and data interpretation as one connected workflow, rather than adding an intelligent layer only after the assay itself has been assembled. Table 6 compares recent SDA platforms developed for portable detection, regenerative nanomachines, molecular logic, machine-learning-assisted analysis, and smartphone or self-powered sensing, highlighting differences in sensitivity, assay duration, and practical applicability.

## 6. Conclusions, Current Challenges, and Future Perspectives

SDA has moved well beyond its original role as a stand-alone isothermal amplification method and now serves as a flexible molecular engine for chemical and biosensing. Strand-displacing polymerases, together with nicking or primer-regeneration reactions, continuously generate programmable DNA outputs without repeated thermal cycling. This reaction architecture readily supports coupling with fluorescence, electrochemistry, ECL, SERS, CRISPR, microchip electrophoresis, lateral-flow devices, and DNA nanomachines [24,26,37,52]. Its analytical scope now extends well beyond nucleic acids. Aptamers, DNAzymes, antibodies, and enzyme-responsive probes can translate proteins, toxins, pesticides, metal ions, and enzymatic activities into DNA triggers that subsequently enter SDA [101,115,122,143]. This modularity suits compact sensing especially well, allowing amplification and signal transduction to be developed independently while remaining readily coupled within the same analytical platform.

The impressive detection limits reported across biomedical, food, and environmental applications should, however, be interpreted cautiously. Attomolar or even lower responses are frequently achieved through cascaded amplification, CRISPR cleavage, nanomaterial enhancement, or secondary DNA circuits rather than SDA alone [58,95,124,155,156]. More importantly, analytical performance in purified targets does not necessarily survive contact with blood, milk, plant extracts, wastewater, or other chemically complex matrices. Recovery loss, nonspecific adsorption, nuclease activity, polymerase inhibition, surface fouling, and uncontrolled background amplification can become more consequential than nominal sensitivity [4,20,21,22]. Multistage designs bring their own practical burdens, including enzyme compatibility, temperature switching, background leakage, cumulative assay time, magnetic handling, electrode fabrication, and lot-to-lot reproducibility. Consequently, a shorter and more robust assay may ultimately carry greater translational value than another order-of-magnitude reduction in LOD.

Comparisons with routinely deployed isothermal tests help place the translational status of SDA in perspective. There is already evidence that the chemistry can move beyond laboratory demonstrations. In the Becton, Dickinson and Company (BD) ProbeTec™ system, target preparation, SDA, fluorescence detection, and automated result interpretation were combined within a regulated molecular diagnostic system [174,175,176]. SDA has shown considerable analytical flexibility, but its commercial presence remains comparatively limited. Loop-mediated isothermal amplification (LAMP) has developed a broader supporting ecosystem, including dedicated reagents, assay kits, primer-design software, compact instruments, and tests for clinical, food, and environmental applications [54,177,178]. Helicase-dependent amplification (HDA) and nicking enzyme amplification reaction (NEAR) have followed another path, with their chemistries incorporated into standardized automated and point-of-care diagnostic systems [179,180].

The commercial limitations of SDA are not primarily a sensitivity problem. They stem more from the difficulty of standardizing a reaction that relies on several interdependent molecular components. Polymerase activity must remain synchronized with nicking or restriction enzymes, and primer–template design has to preserve the sequence features required for repeated recognition and strand regeneration. These requirements complicate enzyme formulation, reagent stabilization, multiplex optimization, and lot-to-lot control. Background amplification and trigger leakage become still more difficult to manage when SDA is coupled to secondary amplification circuits, CRISPR systems, nanomaterials, or molecular machines. LAMP also faces primer-design complexity and nonspecific amplification, but commercial development has progressively packaged these constraints into standardized one-vessel reagents, dedicated instruments, and validated workflows. The comparison therefore suggests that the commercialization gap is primarily a problem of reaction simplification, workflow control, reagent stability, and manufacturability rather than amplification capability alone [54,105,174,177,178]. Table 7 places SDA in the broader commercial landscape of isothermal amplification, comparing its translational strengths and limitations with those of LAMP, HDA, and NEAR in established diagnostic workflows.

Future SDA development should therefore move toward reaction simplification and physical integration. Closed microfluidic cartridges capable of combining extraction, enrichment, amplification, washing, and detection would reduce contamination and operator intervention [9,16,17,18,19]. Paper and porous substrates offer inexpensive passive fluid handling, while near-digital iSDA demonstrates that spatially resolved amplification and smartphone imaging can improve signal-to-noise ratios without the complexity of conventional digital microfluidics [157,172]. For SDA to move beyond controlled laboratory use, reagents must tolerate storage and transport without losing activity. Lyophilizing primers, polymerases, nickases, CRISPR components, and internal controls offers a practical way to reduce that dependence on tightly managed conditions [117,172]. Multiplex SDA leaves little room for cross-interference. Primer sets and nicking sites must work cleanly in parallel, while controls and calibration should remain trustworthy even when the sample matrix changes.

A parallel challenge is to ensure that increasingly intelligent readout improves analytical reliability rather than simply adding computational complexity. Molecular logic, smartphone analysis, and data-assisted multimodal interpretation may sharpen analytical decisions, provided that assay design is robust, calibration data reflect real-world variability, and performance is independently validated across realistic sample and manufacturing conditions [20,22,167,169].

**Table 7:** Commercial maturity and translational characteristics of SDA and representative isothermal amplification methods.

Product/Model/Ref.	Manufacturer/Country	Type	Application	Key Advantages/Limitations
BD ProbeTec™ *Chlamydia trachomatis* (CT); Cat. No. 442959/[174,175,176]	Becton, Dickinson Comp. (BD), USA	SDA	*C. trachomatis* DNA detection	Standardized SDA reagents and automated workflow/requires dedicated BD instrumentation.
BD ProbeTec™ *Neisseria gonorrhoeae* (GC); Cat. No. 442842/[174,175]	Becton, Dickinson Comp. (BD), USA	SDA	*N. gonorrhoeae* DNA detection	Automated SDA and fluorescence readout/limited to the ProbeTec/Viper ecosystem.
BD Viper™ LT System; Model 442839/[175,176]	Becton, Dickinson and Company (BD), USA	SDA	Automated SDA-based nucleic-acid testing	Automated sample processing, amplification, detection, and reporting/instrument-dependent and not exclusively SDA-based.
BD Viper™ LT System SDA Accessory Kit; Model 442958/[175,176]	Becton, Dickinson and Company (BD), USA	SDA	SDA consumables for the Viper LT system	Supports standardized SDA operation on Viper LT/accessory-only and requires compatible instrument/reagents.
Loopamp LF-160 (M-L400)/[177]	Eiken Chemical/Japan	LAMP	Professional *M. tuberculosis* complex detection	High-capacity professional LAMP testing/Target-specific professional use.
Alethia^®^ Incubator/Reader (610189)/[178]	Meridian Bioscience/USA	LAMP	Clinical testing for bacterial, viral, STI, and malaria targets	Portable open field-testing platform/requires external sample preparation.
SOLANA™ Instrument (20278)/[179]	QuidelOrtho/USA	HDA	Clinical molecular testing for respiratory pathogens	Simple HDA-based clinical testing/limited current availability.
ID NOW™ Instrument (NAT-000; NAT-024) /[180]	Abbott Diagnostics Scarborough/USA	NEAR	Rapid near-patient respiratory and Strep A testing	Rapid, compact near-patient molecular testing/low throughput; proprietary consumables.

The next meaningful advance will lie not in greater amplification, but in how completely SDA can deliver a true sample-to-answer workflow. Platforms that com-bine stabilized reagents, enclosed fluid handling, self-powered or low-power trans-duction, internal quality control, digital interpretation, and manufacturable cartridges are likely to define the next stage of SDA sensing [59,134,156,169,171]. Achieving that transition will require standardized performance reporting, larger clinical and field cohorts, longer-term storage studies, manufacturing quality control, cost analysis, and direct benchmarking against PCR, chromatography, sequencing, or established im-munoassays. SDA already possesses the molecular flexibility needed for decentralized chemical sensing; the remaining challenge is to convert that flexibility into reproduci-ble analytical systems that function reliably beyond the laboratory.

## Figures and Tables

**Table 1:** Fundamental, analytical, and implementation characteristics of SDA and PCR for molecular diagnostic applications.

Characteristic	SDA	PCR
Principle	Isothermal polymerase-driven strand displacement with strand regeneration [4,12]	Thermal cycling of denaturation, annealing, and extension [7,8,9]
Temperature	Constant, typically ~37–65 °C [11,12]	Cyclic, typically ~95/50–65/72 °C [7,8,9]
Assay time	Usually ~20–90 min [11,12]	Typically ~1–3 h [7,8,9]
Instrumentation	Simple heating; readily miniaturized [11,12]	Programmable thermocycler required [7,8,9]
Biomedical use	Biomarkers, mutations, pathogens, and recognition-converted analytes [11,12]	Broad molecular diagnostics, genotyping, and gene-expression analysis [7,8,9]
Advantages	Low thermal demand; compatible with portable and microfluidic formats [4,11,12]	Mature, standardized, and quantitative workflows [7,8,9]
Limitations	Background amplification, reaction leakage, and enzyme/design constraints [12,15]	Higher thermal, power, and hardware requirements [7,8,9]
Suitable implementation	Point-of-care, field, disposable, and integrated sensing [4,11,12]	Centralized or well-equipped laboratory testing [7,8,9]
Analytical role	Isothermal polymerase-driven strand displacement with strand regeneration [4,12]	Thermal cycling of denaturation, annealing, and extension [7,8,9]

**Table 2:** Mechanistic comparison of classical SDA and representative SDA design improvements.

SDA Architecture	Modification from Classical SDA	Analytical Principle	Main Benefit	Main Constraint
Classical SDA [2,23]	Repetitive nicking and strand-displacing polymerization	Nicking generates 3′-OH termini; polymerase extension regenerates the nicking site	Continuous isothermal amplification	Enzyme dependence and background amplification
Programmable-output SDA [25,26]	Functional sequences encoded in displaced strands	Products serve as reporters, primers, G-rich strands, or activators	Adaptable downstream readout	Greater sequence-design complexity
Cascaded SDA [12,33,34,35]	SDA products trigger secondary amplification	Primary SDA gain feeds CHA, HCR, primer-exchange, or related circuits	Higher signal gain and flexible conversion	Longer workflows and greater leakage risk
SDA–CRISPR [36,37,38,39,40,41,42,43,44,45]	SDA products activate CRISPR nucleases	Amplified activators initiate reporter cleavage	Sequence-selective amplified readout	Enzyme compatibility and background activation
Branched/multi-output SDA [36,37,38,39]	Multi-arm structures generate several functional strands	Different products activate parallel molecular pathways	Multiplex and coordinated signal processing	Cross-interaction and design complexity
Interfacially confined SDA [46,47,48,49,50,51,52]	Amplification localized at sensing interfaces	Spatial confinement concentrates products near the transducer	Stronger local signal and improved coupling	Surface effects and fabrication variability
Structure-gated SDA [53]	Target-dependent structure controls amplification initiation	Conformational change exposes the productive reaction state	Lower premature amplification and background	Slower kinetics and demanding gate design

**Table 4:** Reported analytical characteristics of representative SDA-based assays for biomedical genotyping, epigenetic analysis, proteins, enzyme activities, and viral targets.

Detection Target	Detection Mode	Active Substance/Sensing Element	Reported SDA Condition/Time	Reported Detection Range and LOD	Ref.
*PIK3CA* H1047R SNV	Label-free FL	FspI; KF; Baby Spinach RNA aptamer/DFHBI-1T	SDA: 37 °C, 150 min	R: 10 fM–1 nM, LOD 95 aM.	[91]
Dam MTase	FL	Bifunctional hairpin DNA (bHpDNA); CRISPR/Cas12a + FAM reporter	SDA at 37 °C, 80 min	R: 0.25–1.25 U/mL, LOD 2.458 × 10^−3^ U/mL	[95]
Septin 9 methylation	Label-free FL/LFA	GlaI; dual SDA;G31 G-triplex/ThT	DCSDA: 37 °C, 60 min; Cas12a: 37 °C, 40 min	R: 0.2 pM–200 nM, LOD: 170.66 fM	[98]
KRAS G12C mutation	FL	FEN1 for mutation; KF	SDA: 37 °C, 30 min	10 nM synthetic T1/T2	[100]
CEA	PEC	Aptamer, MB, 3D DNA nanosphere, ZnSe QDs/AuNPs	Enzyme-free SDA, RT, 2 h	R: 1 fg/mL–10 ng/mL, LOD 0.12 fg/mL	[103]
EBV LMP-1 protein	Amperometric	LMP-1 Ab, TDN/AuE, Ab-AuPt nanozyme	SDA: 53 °C, 45 min;	R: 0.1–1500 pg/mL, LOD 47 fg/mL	[104]
T4 PNKP	FL	Cas12a/crRNA, FQ probe	Klenow/Nb.BbvCI, SDA: 37 °C, 50 min	R: 10^−5^–2.5 × 10^−2^ U/mL, LOD 3.3 × 10^−6^ U m/L	[107]
Dam MTase	SERS	HAuE, KFP, Nb.BbvCI, AuNCs/4-MBA	SDA: 37 °C, 2 h	10^−4^–0.5 U/ mL, LOD 8.65 × 10^−5^ U/mL	[108]
Dam MTase	FL	Nt.AlwI, Klenow, SG	CSDA: 37 °C, 30 min	R: 2–40 U/mL, LOD 0.67 U/mL	[109]
FTO (m^6^A demethylase)	FL	m^6^A recognition probe, BtsI, poly(A) primer, SYBR Gold	HSDA: 37 °C, 60 min	R:3.0 × 10^−10^–1.0 × 10^−7^ mg/mL, LOD 2.6 fM	[115]
Largemouth bass virus	Lateral-flow/visual	LA38s aptamers, Klenow fragment, AuNP-DNA probe	SDA: 37 °C, 30 min	R: 8 × 10^1^–8 × 10^7^ cells/mL, LOD 8 × 10^1^ cells/mL	[118]
SARS-CoV-2 N-RNA	FL	Anti-NP-P1/P2, blocker, KF	SDA: 37 °C, 20 min	R: 100 fM–1 nM, LOD 5.5 fM	[120]
SARS-CoV-2 ORF1ab/N	FL	L1/L2, KF, Nt.BbvCI, Cas12a/crRNA	SDA + CRISPR; CRISPR 30 min	ORF1ab: LOD 1.0 aM; N: 0.9 aM; AND: 10 aM–20 nM/3.7 aM	[121]

CEA: carcinoembryonic antigen; CSDA: cascade SDA; DCSDA: double-cascade SDA; EC: electrochemistry; HSDA: hyperbranched SDA; LFA: lateral flow assay; MTase: methyltransferase; Nt.BbvCI: nicking Endonuclease BbvCI; OTA: Ochratoxin A; SERS: surface-enhanced Raman scattering; ThT: Thioflavin T.

**Table 5:** Representative SDA sensing platforms for food, environmental, and field-oriented applications.

Detection Target	Detection Mode	Active Substance/Sensing Element	Reported SDA Condition/Time	Reported Detection Range and LOD	Ref.
ZEN	DPV EC	Multifunctional hairpin, PDANSs, MB/ITO	SDA: 37 °C, 90 min; exo-Klenow + Nb.BbvCI	R: 1–150 pg/mL, LOD 0.18 pg/mL	[122]
Biotin	FL	SA–biotin/Klenow + Nb.BbvCI/Cas12a	SDA: 37 °C, 40 min	LOD 0.01 nM	[127]
CAP	ECL	SDA-induced bipedal DNA walker, ordered DNA tracks	SDA: 37 °C, 2 h; KF + Nb.BbvCI	R: 1 fM–100 nM, LOD 0.71 fM	[128]
*mecA*	PT-LFTS	CS NPs/Bst + Nb.BbvCI/CSA-DNA	LD-RPA-SDA, ≤30 min	R: 3.9–3.9 × 10^6^ copies/μL, LOD 0.43 copies/μL	[130]
*S. aureus*	ECL	Fe_3_O_4_/aptamer:T, essential-oil-synthesized	SDA: capture 60 min; walker reaction 60 min	R: 10^2^–10^7^ CFU/mL, LOD 17 CFU/mL	[131]
*L. monocytogenes*	EC-DPV	Apt-MBs/SDA-RCA-HCR/SA-HRP	SDA: 37 °C, 60 min	R: 15–1.5 × 10^8^ CFU/mL, LOD 5 CFU/mL	[133]
Cr^3+^	Ratiometric UCL	DNAzyme/UCNPs–TAMRA	SDR: 37 °C, 40 min	R: 0.5–75 nM, LOD 0.135 nM	[142]
Hg^2+^	ECL	phi29, Nt.BbvCI/Ce_2_Sn_2_O_7_–P5FIn	SDA: 37 °C, 2 h	R: 1 fM–100 nM, LOD 0.30 fM	[144]
*bla*-TEM, *tetM*, *sul-1*, *bla*-CTX-M-1	FL	TCPE–DNA hydrogel/3D microextractor	SDA: 35 °C, 90 min	R: 1 fM–100 nM, LOD 0.159 fM	[147]
*bla*-TEM	SERS	KF polymerase, Nt.BbvCI/AuNPs–MBs	SDA: 37 °C, 1 h	R: 0.1 zM–300 aM, LOD 0.02 zM	[149]
SGIV-infected cells	Lateral flow/colorimetry	Aptamers, Klenow exo^−^, Nt.BbvCI, AuNPs	SDA: 37 °C, 30 min	R: 1 × 10^4^–5 × 10^6^ cells/mL, LOD 5 × 10^4^ cells/mL	[153]
CaMV 35S promoter	FL	Klenow, Nt.BbvCI, CRISPR/Cpf1	SDA: 37 °C, 100 min	R: 50 fM–500 pM, LOD 14.4 fM	[154]
Pokkah boeng disease DNA	EC	DNA walker, Phi29, Nb.BbvCI, AuNPs/S-GDY	Dual SDA, 60 min	R: 0.1 fM–10 nM, LOD 16.6 aM	[155]
*S. scitamineum* DNA	EC	CRISPR/Cas12a, Phi29, Nt.BbvCI, Fe_3_O_4_@AuNPs/B-GDY	SDA, 60 min	R: 0.0001–100 pM, LOD 41.56 aM	[156]

B-GDY: boron-doped graphdiyne; CaMV 35S: cauliflower mosaic virus 35S promoter; CAP: Chloramphenicol; COF: covalent organic framework; CSA: gold-coated copper selenide nanoparticles; EDC: entropy-driven catalysis; LD-RPA-SDA: light-driven-RPA-SDA; LFTS: lateral-flow test strip; SA-HRP: Streptavidin–horseradish peroxidase; S-GDY: sulfur-doped graphdiyne; SGIV: Singapore grouper iridovirus; UCL: upconversion luminescence; ZEN: zearalenone.

**Table 6:** Representative AI-enabled, intelligent, and digital RCA platforms for diagnostic interpretation, molecular logic, automation, and quantitative analysis.

Detection/Computational Model	Reported Performance and Analytical Contribution	Assay Architecture/Total Time	Target and LOD	Ref.
Distance-based visual	Naked-eye quantitative distance readout Low-cost paper format for POCT Serum results agree with qRT-PCR	Paper-based; SDA/hydrogel 60 min + readout 10 min	let-7a; 1 fM	[48]
AuNP-LFA	Extraction-free multiplex viral detection Naked-eye readout without instrumentation Freeze-dried reagents support on-site testing	LFA; RT-SDA 30 min + LFA 10 min	Influenza A/B, SARS-CoV-2; 20/63/46 copies/μL	[117]
Aptamer-AuNP LFA	Aptamers enable selective viral capture Magnetic enrichment improves target isolation Suitable for field aquaculture surveillance	LFA; total ~60 min	LMBV; 8 × 10^1^ cells/mL	[118]
Photothermal	Thermometer replaces laboratory spectrometers AuNP aggregation enables thermal conversion Clinical cervical samples successfully validated	Portable photothermal; ~3 h	HPV16; 0.3 fM	[159]
MCE FL	Protein recognition-converted into ssDNA Low sample and reagent consumption Serum analysis demonstrates practical applicability	Microchip-based; SDA 2 h + MCE ~3 min	MUC1; 0.23 pg/mL	[160]
FL/FDD	Three autonomous amplification cycles enhance sensitivity Single probe simplifies molecular reaction architecture Enables point-mutation and cellular RNA analysis	Self-driven intelligent nanomachine; SDA: 37 °C, 80 min	miR-21; 0.479 fM	[162]
FL	SDA-derived NEAA regenerates trigger strands One-pot design reduces manual reaction steps Detects UDG activity from HeLa lysates	Regenerative NEAA–APE1 one-pot system; SDA: 37 °C, 6 h	UDG activity; 1 × 10^−5^ U/mL	[164]
EC/colorimetric/PT + ML	Triple-modal outputs enable internal cross-validation Elastic Net automates multimodal signal calibration Self-powered operation supports decentralized field analysis	Data-driven: SDA–Exo III + EBFC + Elastic Net	Pokkah boeng DNA; 16.92 aM/21.75 aM/2.86 fM	[169]
CRISPR logic	AND logic enables dual-RNA molecular decisions DTN interface improves Cas12a cleavage accessibility Particle counting suppresses bulk fluorescence background	Molecular logic: Cas13a–SDA–Cas12a; <90 min	HPV E6/E7 mRNAs; 485 aM	[170]
Self-powered EOCV/smartphone	Capacitor increases current sensitivity 10.6-fold Smartphone enables wireless real-time signal acquisition Flexible EBFC removes external power dependence	Self-powered integration: dSDA–HCR + EBFC; 180 min	miR-21; 32.3 aM; capacitor 7.1 aM	[173]

AuNP: Gold nanoparticle; EBFC: enzyme biofuel cell; EOCV: open-circuit voltage; HPV16: human papillomavirus type 16; LFA: lateral-flow assay; LMBV: largemouth bass virus; MCE: microchip electrophoresis; ML: Machine learning; MUC1: Mucin 1; POCT: point-of-care testing; RT-SDA: reverse transcription SDA.

## Data Availability

No new data were created or analyzed in this study. Data sharing is not applicable to this article.
